# Supplementary material for: Genome-level selection in tumors as a universal marker of resistance to therapy
Source: Nat Commun. 2025 Jul 16;16:6535. doi: 10.1038/s41467-025-61709-x (PMC12263839; doi:10.1038/s41467-025-61709-x)
Supplement: Supplementary file 1 — Supplementary Information [file 41467_2025_61709_MOESM1_ESM.pdf]

Supplementary Information

Supplementary Figures

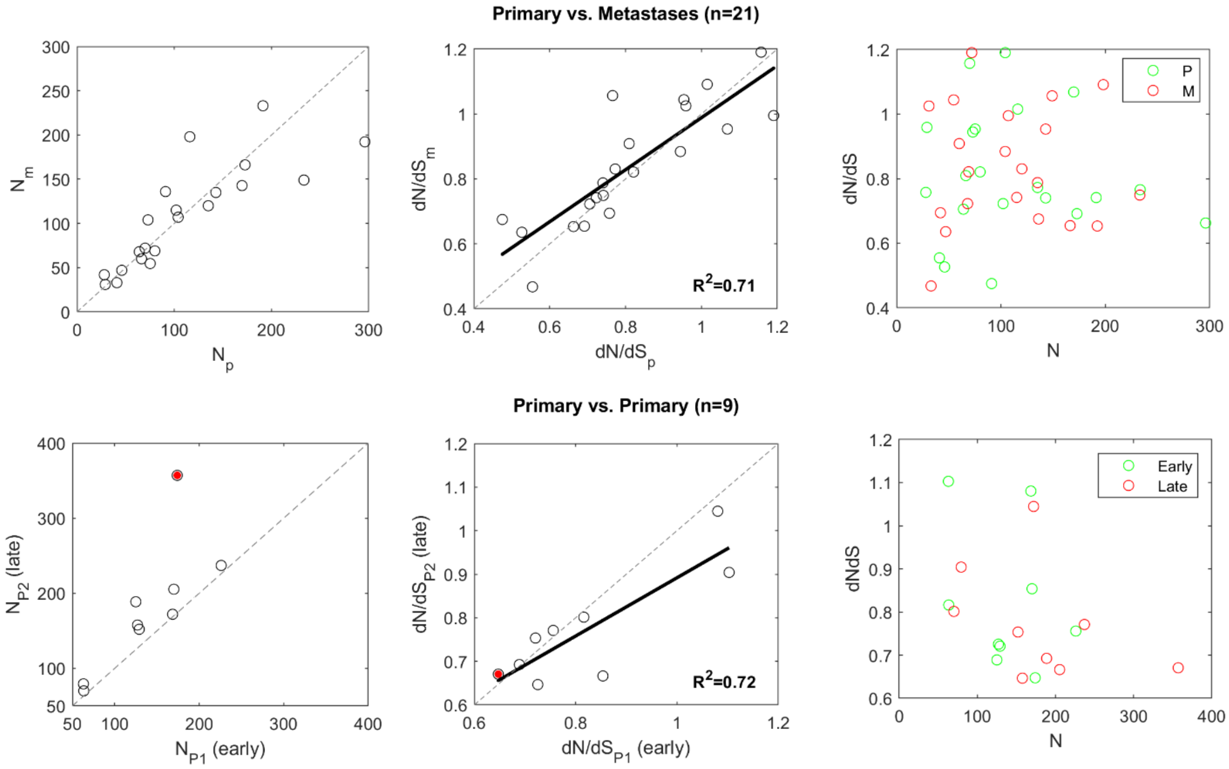

Figure S1: **Analysis of untreated colorectal cancer patients. Upper panel**) the relationship between the average non-silent mutations *N* (***left***), the average selection *dN/dS* (***middle***), of primary (P) samples and metastases (M) samples, in each of the 21 patients (out of 23) that contain untreated samples in both P and M states. The relationship between *dN/dS* and *N* in each of the states is also shown (***right***), indicating a non-monotonic behavior, with a peak of *dN/dS* in intermediate *N* (~100). *dN/dS* increases with *N* at low *N* as a sign of relaxed or positive selection (presumably of driver mutations) and decreases with *N* for large *N* as a sign of purifying (negative) selection (presumably of deleterious passenger mutations). Note that this non-monotonic behavior exists in both P and M states. **Lower Panel**) Similar comparisons are shown, of *N* (***left***) and *dN/dS* (***middle***), between early (P1) and late (P2) primary samples in each of the 9 patients that contain multiple primary samples. Note that big changes in *N* (red mark) do not necessarily affect the *dN/dS* invariance. The relationship between *dN/dS* and *N* is also shown (***right***). As most points are above the intermediate critical *N* (of ~ 100) only the decrease of *dN/dS* with *N* is notable.

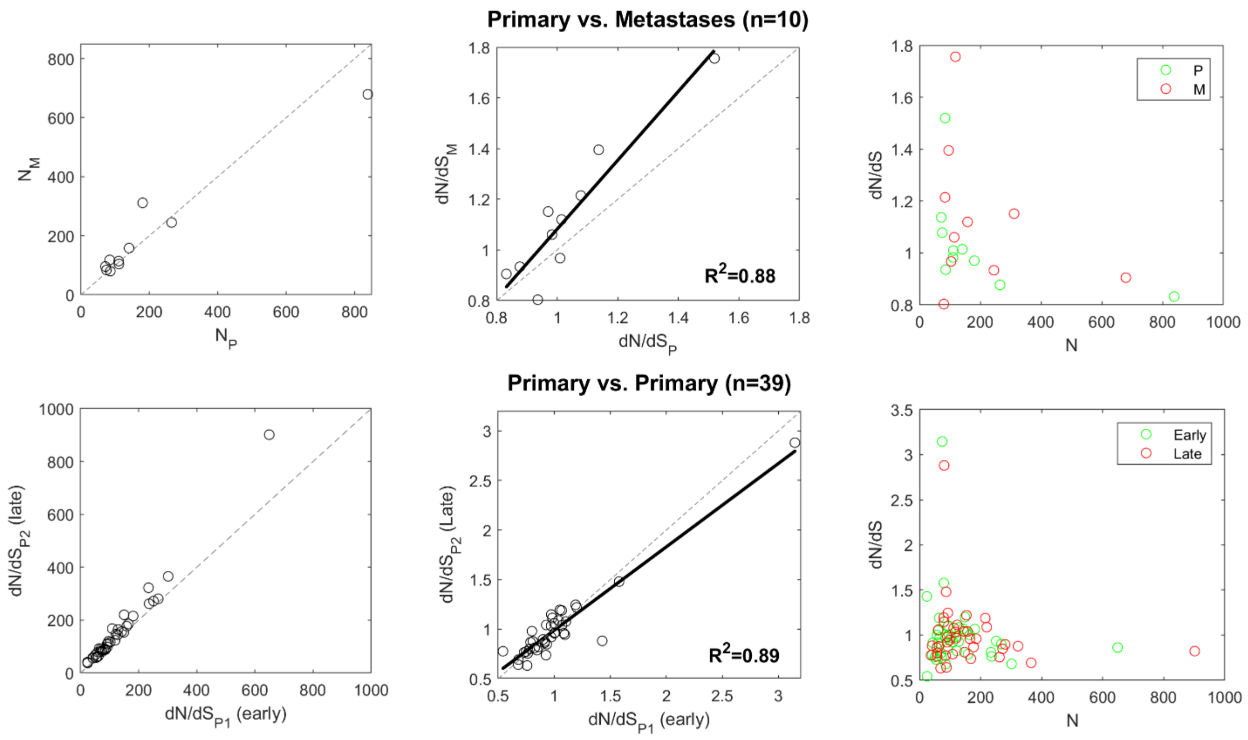

Figure S2: **Analysis of untreated esophageal cancer patients. Upper panel**) the relationship between the average non-silent mutations *N* (***left***), the average selection *dN/dS* (***middle***), of primary (P) samples and metastases (M) samples, in each of the 10 patients that contain untreated samples in both P and M states. The relationship between *dN/dS* and *N* in each of the states is also shown (***right***), indicating a non-monotonic behavior, with a peak of *dN/dS* in intermediate *N* (~100). *dN/dS* increases with *N* at low *N* as a sign of relaxed or positive selection (presumably of driver mutations) and decreases with *N* for large *N* as a sign of purifying (negative) selection (presumably of deleterious passenger mutations). Note that this non-monotonic behavior exists in both P and M states. **Lower Panel**) Similar comparisons are shown, of *N* (***left***) and *dN/dS* (***middle***), between early (P1) and late (P2) primary samples in each of the 39 patients that contain multiple primary samples. The relationship between *dN/dS* and *N* is also shown (***right***). The non-monotonic behavior with a peak at intermediate N is notable also here and holds for both early and late primary samples.

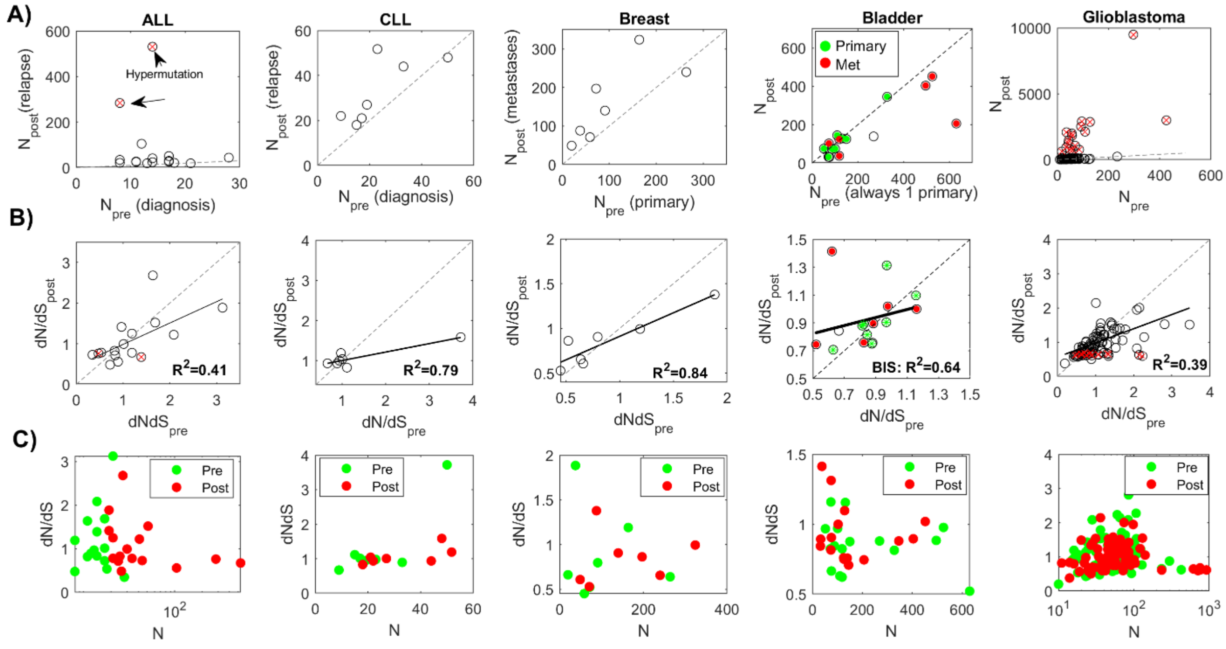

Figure S3: **Evolutionary status of treated cancers. A**) The relationship between the number of *N* mutations before and after therapy for the 5 studies examined (*cf.* **Figure 2** of the main text): ALL under chemotherapy, CLL under BCL-2 inhibition, Breast ER-positive under Aromatase inhibitors, Bladder under chemotherapy and Glioblastoma under radiology and alkylating agents. In most cases, except for the bladder cohort, the tendency is for higher number of mutations post-therapy. Cases of hyper mutations (in ALL and Glioblastoma cohorts) are marked (red star). **B**) the relationship between *dN/dS* values before and after therapy as depicted in **Figure 2** of the main text are shown again for convenience. **C**) the relationship between *dN/dS* and *N*, across the 5 cohorts, are shown for all the treatment-naïve samples (Pre; in green) and for all the post-treatment samples (Post; in red). ALL and glioblastoma are shown with the X-axis in the log scale for clarity (due to the hyper mutation cases).

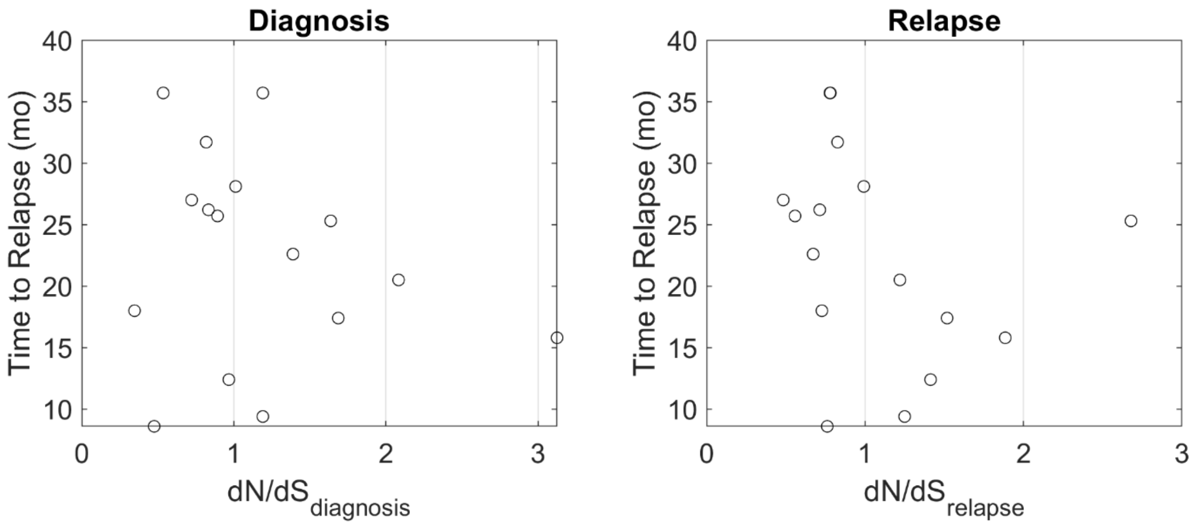

Figure S4: **Time to relapse versus the *dN/dS* values in the ALL cohort. Left**) The time to relapse in months (mo) versus the *dN/dS* values at diagnosis prior to chemotherapy. **Right**) The time to relapse versus the *dN/dS* values at relapse after therapy. The values at relapse appear to be correlated with the time to relapse, whereby *dN/dS* values close to neutrality (i.e., close to 1) exhibit short time to relapse. Note the decrease (negative slope) for $dN/dS < 1$ and the increase (positive slope) for $dN/dS > 1$.

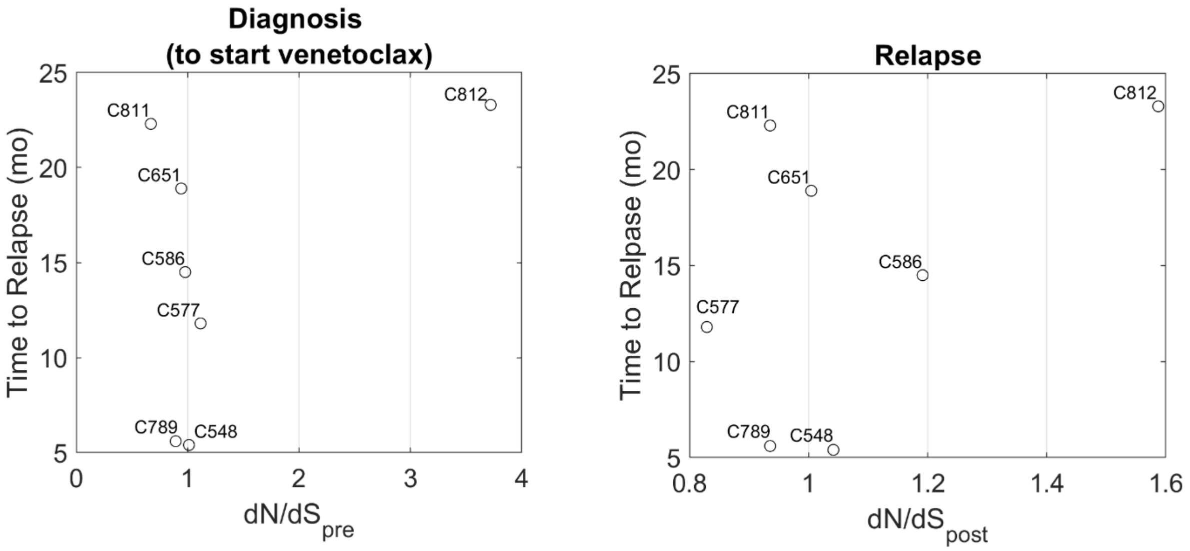

Figure S5: **Time to Relapse versus the *dN/dS* values in the CLL cohort. Left**) The time to relapse in months (mo) versus the *dN/dS* values at diagnosis (for the new targeted therapy of BCL-2 inhibition by venetoclax). Note that all patients in this cohort previously received 1 to 8 lines of standard therapy. **Right**) The time to relapse versus the *dN/dS* values at relapse after the new therapy. Both at diagnosis and relapse *dN/dS* values close to neutrality (i.e., close to 1) have the shortest time to relapse (C789, C548) but those patients that somewhat escape neutrality following therapy do better (C577, C586). The patients with the longest time to relapse (C811, C812) are the most far from the neutral regime, although get closer to it following therapy and eventually relapse as well (see main text). Note that an exception to this is the case C651 that started and remained around *dN/dS≈1*, however, this patient did not respond to the therapy to begin with.

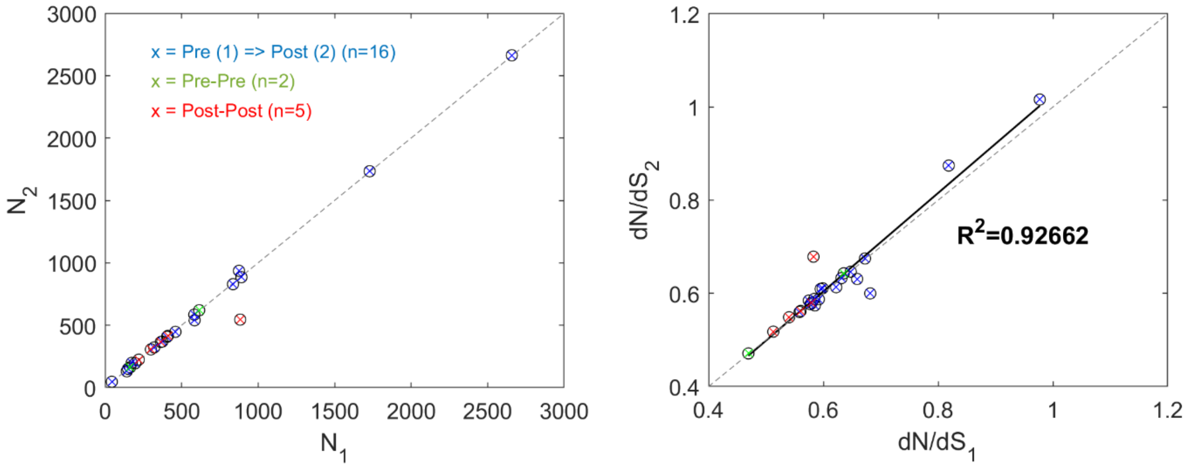

Figure S6: **Evolutionary status of patients in the melanoma cohort.** The melanoma cohort consists of 86 metastatic samples from 53 patients. Most patients were treated with chemotherapy Dacarbazine (DTIC). From this cohort we identified 23 patients with multi-regions (2-5 samples), where 16 patients include pre and post therapy samples, 2 cases with multiple samples pre-treatment and 5 cases with multiple post treatment samples. **Left**) The relationship between the number of *N* mutations of early vs. late sample in each category (color code). **Right**) The corresponding relationship between *dN/dS* values (same color code).

Data Set	Patients	Treatment (at the time of biopsies)	Samples (Set I)	Samples (Set 2)
**1.** Nayar et al, Nat Gen (2019)	7/8 **Breast** (ER+) resistant	**Yes** (ER-targeted)	1 Primary (Pre)	1 distant metastases (post)
**2.** Caswell et al, Nat Com (2019)	5 **Breast** (HER2+)	**Yes** (HER-targeted)	1 Primary (pre)	2-6 Primary (post)
	4 **Breast** (mix)	**No**	1 Primary (core)	1-3 Primary (regions)
**3.** Barry et al, Clinical Cancer Res (2018)	10/11 **Breast** (mix)	**No**	2 Primary (1-6cm apart)	1-5 LN (early; no distant metastases)
**4.** Ng et al, Clinical Cancer Res (2017)	9 **Breast** (mix)	**No** (only after biopsies)	1 primary	1 distant metastases (synchronous)
**5.** Miller et al, Nat Com (2016)	21/22 **Breast** (ER+) sensitive/resistant cases	**Yes** (ER-targeted)	1 Primary (pre)	1 Primary (post, 4 months after therapy)

Figure S7: **List of 5 breast cancer patient cohorts**. The analyzed studies for validation (*cf.* **Figure 3**), span untreated and treated cases, that enable comparisons between regional primary sample across time and between primary and local and distant metastasis, without treatment and before and after treatment. No. of patients indicates the number of quantifiable cases out of (/) the existing ones in the respective studies.

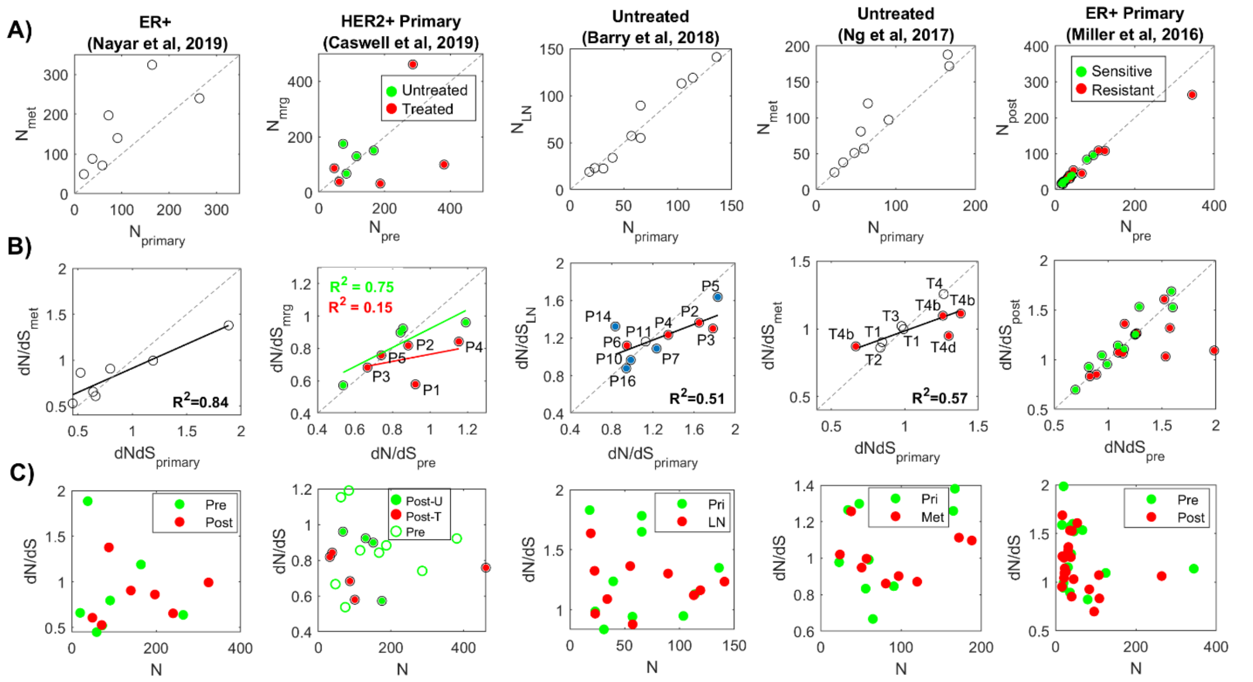

Figure S8: **Analysis of 5 untreated and treated breast cancer cohorts. A)** The relationship between the number of *N* mutations between the two sets compared (*cf.* **Figure S7** and **Figure 3**) across the 5 cohorts. **B**) The respective relationship between the *dN/dS* values of the compared sets across the 5 cohorts. The names of patients correspond to their patient-ID in each respective study. In the study of HER2+ patients (*second column*) patients P1-P5 are treated (red) and the untreated cases are marked as well (green). In the study of untreated early LN metastasis (*third column*), patient P11 was classified as a linear progression (black), while patients P2, P3, P4, and P6 were classified as exhibiting early LN divergence (red), and patients P5, P7, P10, P14 and P16 did not display clear separation of LN from the primary tumors (blue), except for P14 which was heavily driven by APOBEC mutagenesis. Patient P9 was not quantifiable. In the study of distant metastases (*forth column*), the patients in advanced stages, T4b and T4d (red), a shift to neutrality is identified, but not lower stages which exhibit strict linear relationship in between their *dN/dS* value. In the study of distant metastases (*fifth column*), patients that were sensitive to 4mo of neo-adjuvant therapy (green) and those that were resistant (red) are shown, indicating that only (some) resistant cases exhibit a shift to neutrality. **C**) the relationship between *dN/dS* and *N* across the 5 cohorts, for each of the treatment-naïve primary samples (green) and for each of the metastatic samples (treated and untreated) and post-treatment primary (red).

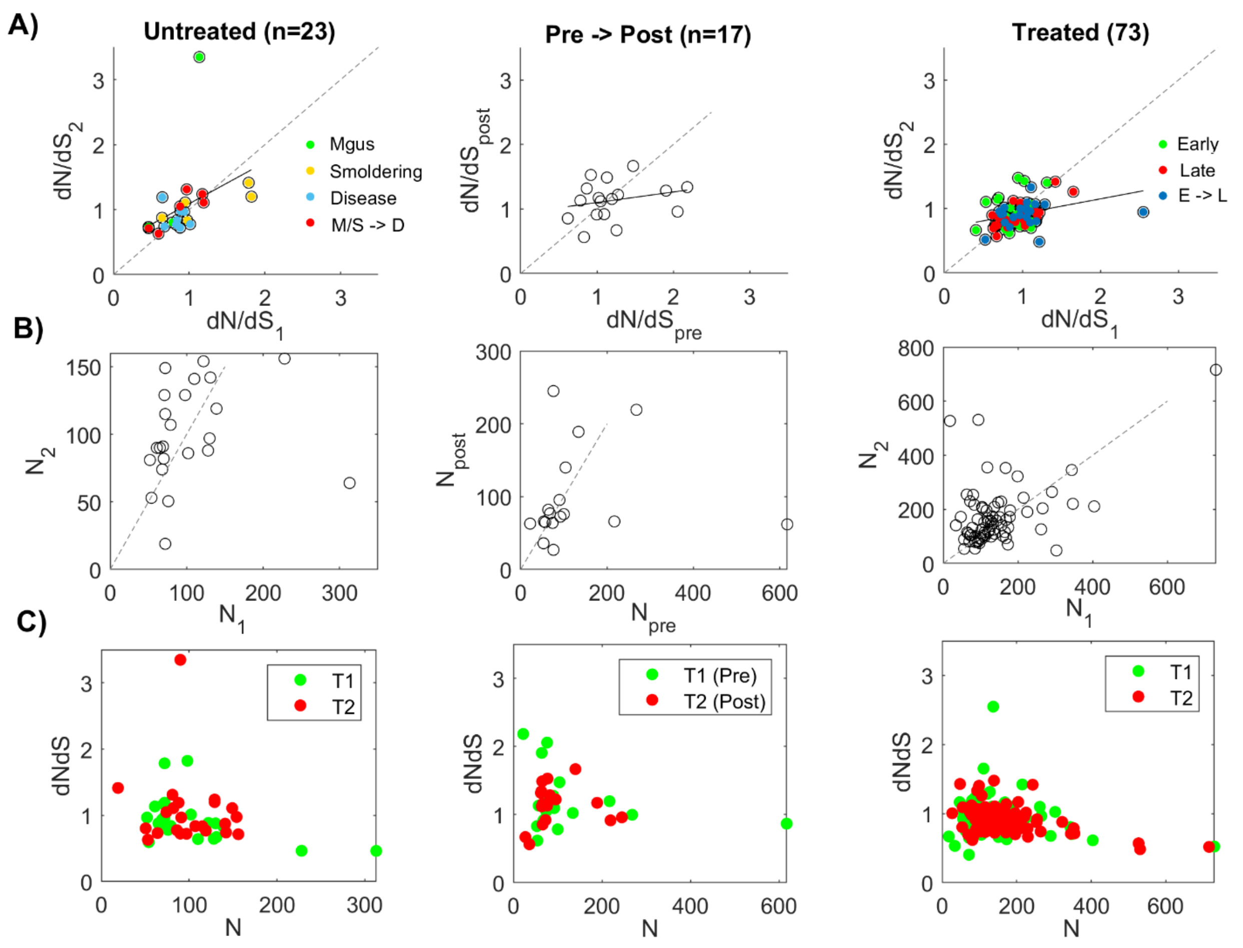

Figure S9: **Evolutionary status of multiple myeloma cohort. A**) The relationship between the *dN/dS* values across patients with multiple samples of the same state and/or of different states of progression (color codes), comparing early samples (1) with later samples (2), shown for: untreated 23 patients (*left*), including MGUS, smoldering, and treatment-naive active state of the disease and the transition from the benign states to the disease state (M/S->D), 17 patients with sample before and after therapy (*middle*), and 73 patients with early (E) and/or late (L) recurrences under treatment (*right*). **B**) The same comparisons between samples across patients as in A are shown for the values of *N* mutations. **C**) The relationship between dN/dS and N for each of each of the samples' groups (i.e., 1 and 2; as in the color code) compared in A and B.

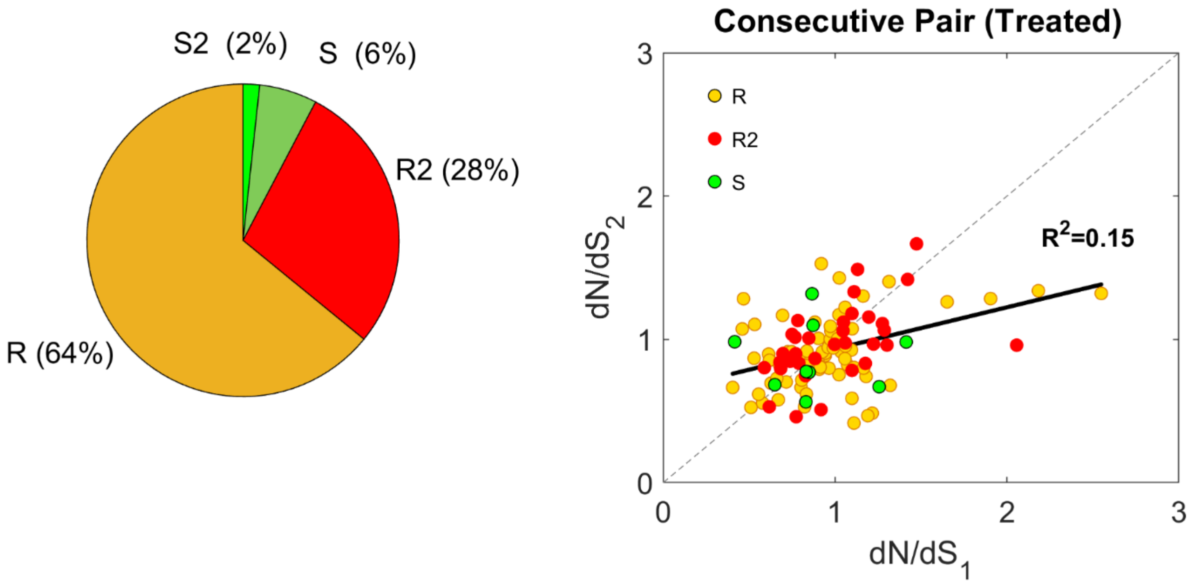

Figure S10: **Response to therapy in the multiple myeloma cohort. Left**) The distribution of the resistant (R) and sensitive (S) cases to the applied therapy, between any two consecutive biopsies, classified into 4 categories: always resistant (R), starts sensitive but becomes resistant (R2), always sensitive (S) and starts resistant but becomes sensitive (S2), as deduced from L/K ratio and M-spike measurements in clinics, depicting that the majority of cases represent resistance to the applied therapies. **Right**) The relationship between the *dN/dS* of two consecutive biopsies, colored by the classification in A, with S and S2 united to one group. Note that some of the therapy-sensitive cases form 'lines of escape' from the neutral regime (in red for R2 category, and green for the S category)

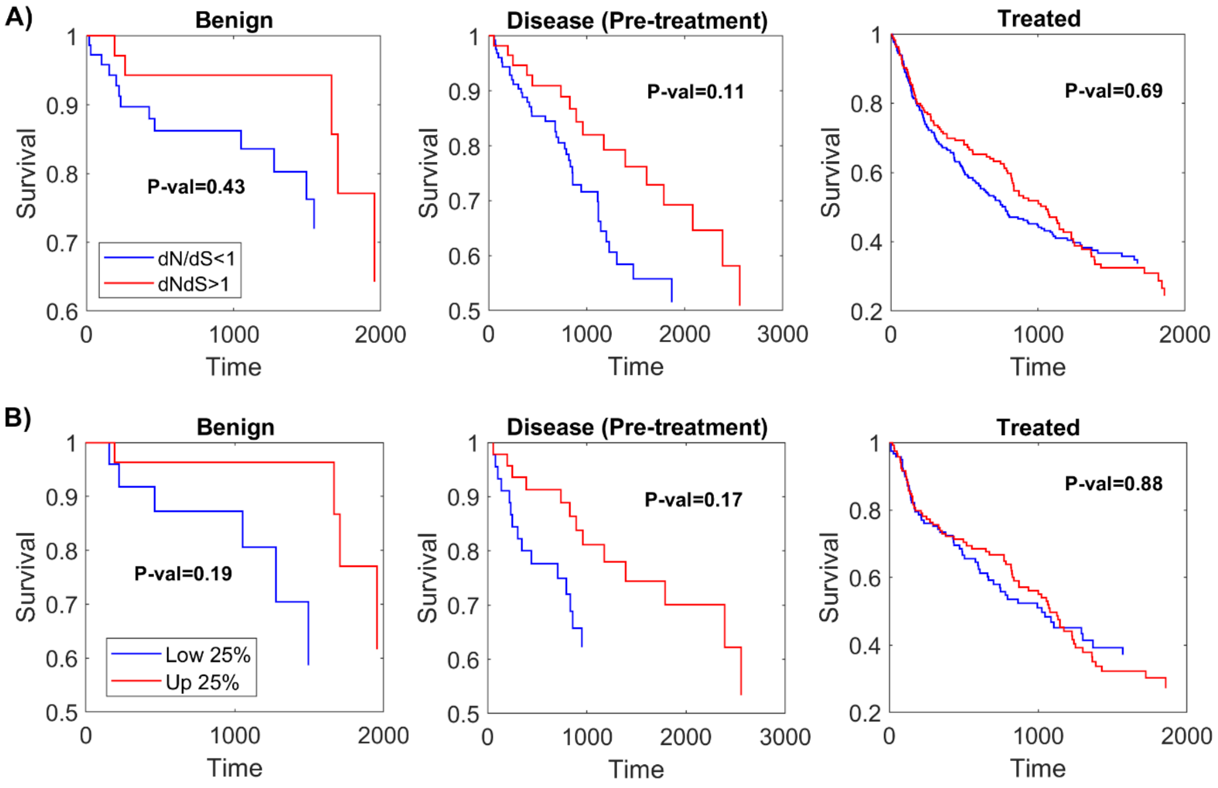

Figure S11: **Kaplan-Meier survival analysis of the multiple myeloma cohort by *dN/dS*.** **A**) Comparison of positive selection (*dN/dS*>1; red) against negative selection (*dNdS*<1; blue) showing no significant results, as indicated by the P-values, across the different phases of the disease (e.g., from benign states to the treated recurrences). **B**) Comparison of extreme positive selection (Upper 25% of *dN/dS* values; red) against extreme negative selection (lower 25% of *dNdS* values; blue) also displaying no significant results, as indicated by the P-values. Note that therefore only the neutral regime (*cf.* **Figure 4C**) is associated with significantly poor prognosis.

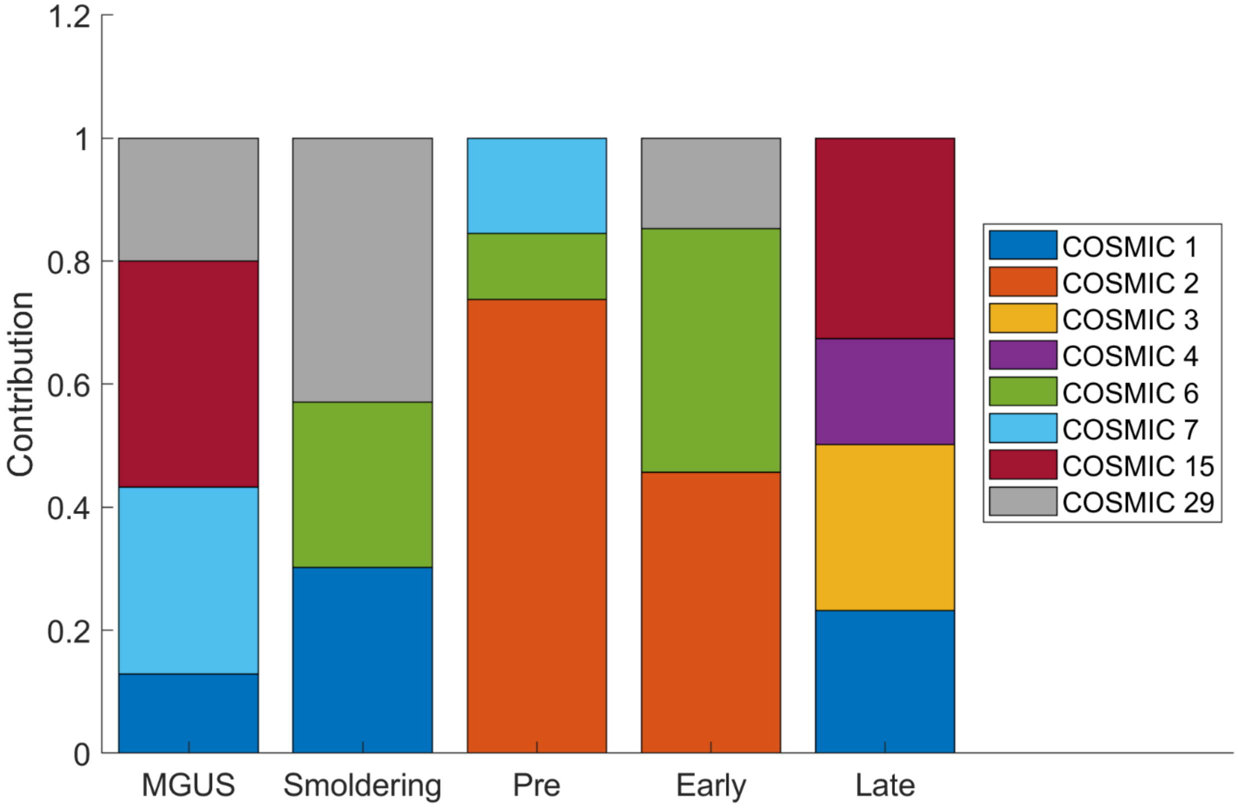

Figure S12: **COSMIC mutational signatures in the multiple myeloma cohort.** The contribution of COSMIC signatures were evaluated in each patient, and the average contributions of each signature across patients are shown, for patients in respective statuses of disease progression. Note that signature 1 (spontaneous deamination of 5-methylcytosine) and 2 (activity of the AID/APOBEC family of cytidine deaminases) are dominant across most cancer types, and the additional dominant signatures in this cohort relate to DNA damage (signature 3, 6,15) tobacco carcinogens (signatures 4, 29) and exposure to ultraviolet (signature 7). These features are also reflected in the analysis of individual patients (cf. **Figure 5A**)

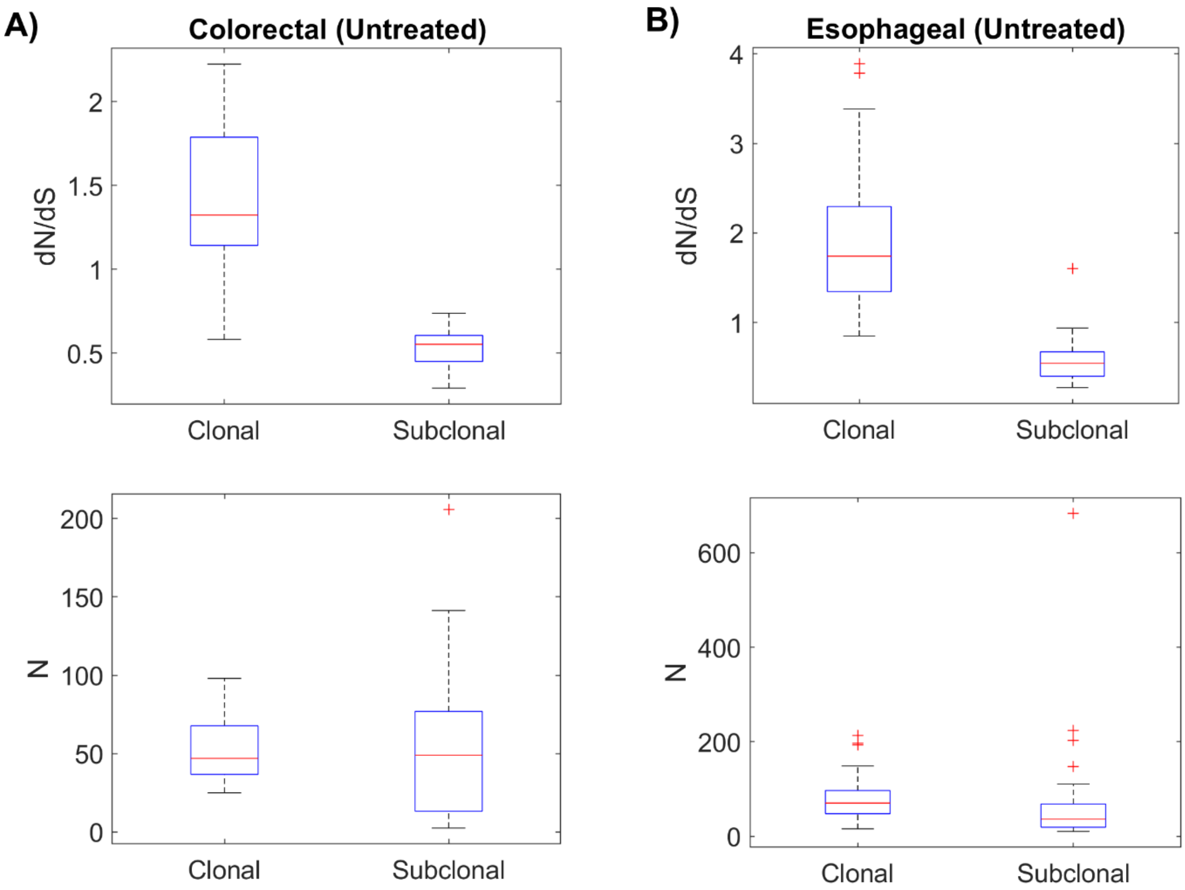

Figure S13: **Evaluation of dN/dS for trunk and branch mutations. A)** a similar analysis to that presented in **Figure 5B** was conducted for the (mostly) untreated colorectal cancer patients. The distribution of dN/dS (***upper***) and N (***bottom***) are shown for mutations at the trunk of the phylogenetic trees of patients, defined as those shared by all samples in a patient (Clonal) and for private mutations, at the branches of tree of a patient (Subclonal). Clonal mutations exhibit elevated dN/dS as a sign of positive selection while subclonal mutations exhibit low dN/dS values as a sign of negative selection. Note that here the length of the trunk and branches are comparable. **B**) a similar analysis of the untreated esophageal cancer cohort. Note that here the trunk is longer than the branches, but the difference in dN/dS between clonal and subclonal mutations is like all other cancers. **Thus, combined with Figure 5B, positive selection in clonal mutations and negative selection in subclonal mutations are universal and independent of the tree shape or treatment status.** Box plots denote the median (red) and the edges of the box are the 25th and 75th percentiles, with whiskers extending to the most extreme data points.

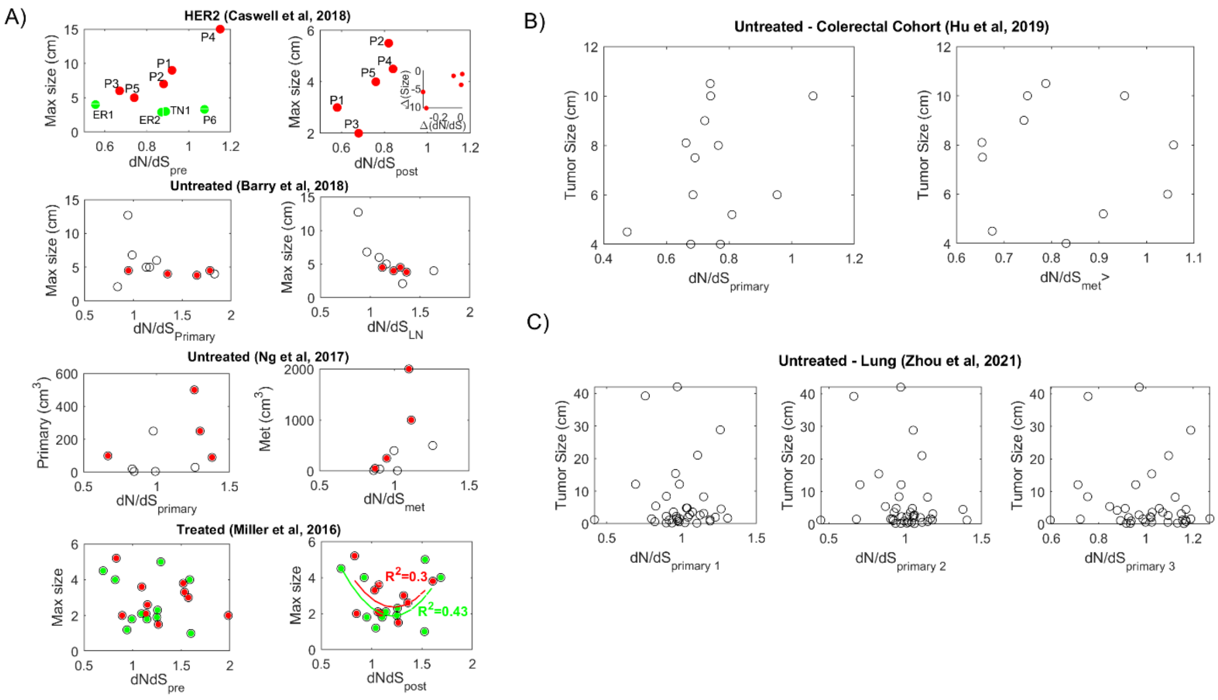

Figure S14: **dN/dS values versus tumor size. A**) The relationship between the *dN/dS* values and the tumor size in 4 out of the 5 breast cancer cohorts (where tumor size is available). From up to bottom: (**1**) in the case of HER2 targeted therapy, the untreated cases (green) show no correlation between the two variables, while in the treated cases (P1-P5, red; *cf.* **Fig. S8B**) there's increase in tumor size as the dN/dS value approached neutrality (i.e., 1) from below, indicating that neutrality is associated with proliferation. Further, the large decrease in tumor size following treatment (post – pre) is associated with decrease in dN/dS (inset). (**2**) in the untreated study of local LN spread a weaker tendency for higher tumor size as dN/dS values approach 1 is observed. Red symbols indicate the early diverged cases (P2, P3, P4 and P6; *cf.* **Fig. 3** and **Fig. S8B**). (**3**) In the untreated study of distant metastases, no significant relationship between the two variables is observed. Red symbols indicate the advanced cases (>T4; *cf.* **Fig. 3** and **Fig. S8B**). (**4**) In the treated study of ER+ cases, there is no significant relationship between pre-treatment dN/dS and the tumor size, however, the dN/dS post-treatment is somewhat correlated with tumor size (R-square of quadratic fit), indicating that in this case, small tumors and drift and maybe linked to the neutral regime. This applies to both sensitive (green) and resistant (red) cases **B) T**he relationship between the *dN/dS* values and the tumor size in the untreated colorectal cancer cohort, showing no significant association between the two variables. **C**) **T**he relationship between the *dN/dS* values and the tumor size in the Untreated lung cancer cohort, showing no significant association between the two variables.

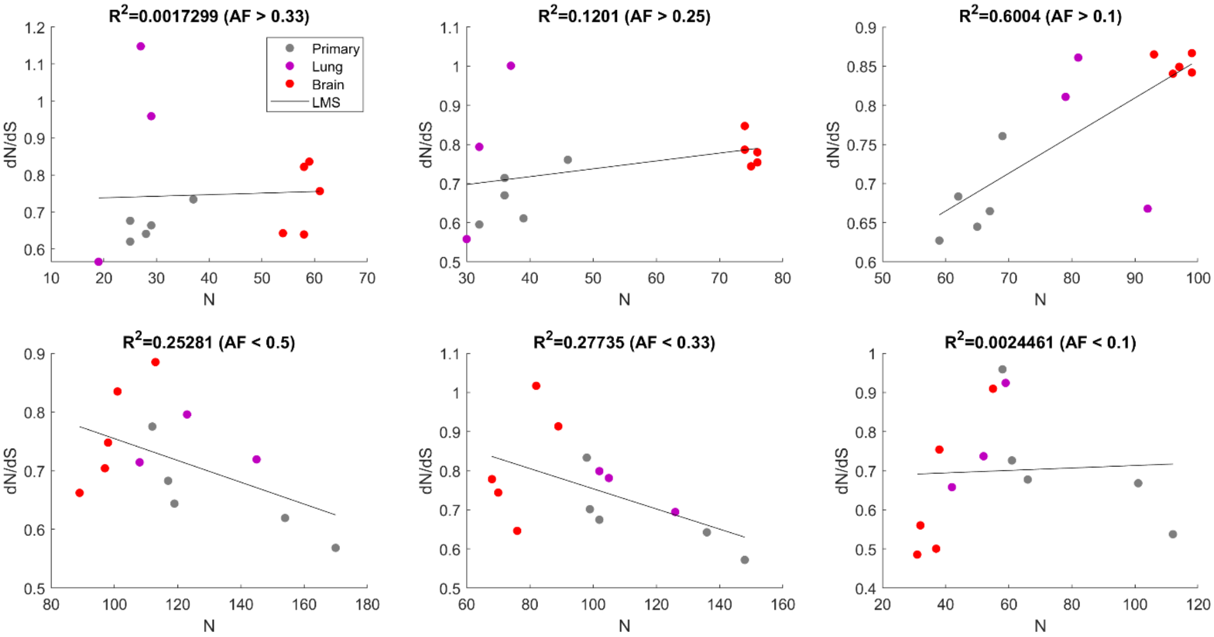

Figure S15: **Analysis of a colorectal patient (V930) with early metastatic dissemination. Upper Panel**) The relationship between *dN/dS* and *N* of 13 samples, including untreated primary (n=5, grey) and post-treatment lung (n=3, purple) and brain (n=5, red) metastasis, for different ranges of AF (clonal-enriched), increasingly including more mutations, from left to right (AF > 0.33, 0.25 and 0.1). A positive correlation is revealed with the increasing AF range and number of mutations. **Lower Panel)** The relationship between *dN/dS* and *N* for different ranges of AF, increasingly focusing on later (subclonal-enriched) mutation mutations (and excluding clonal), from left to right (AF < 0.5, 0.35 and 0.1). A negative correlation is revealed. **Note that** for the untreated primary samples (gray) the positive correlation in the upper panel and the negative correlation in the lower panel are clearer than they are for the treated metastatic samples.

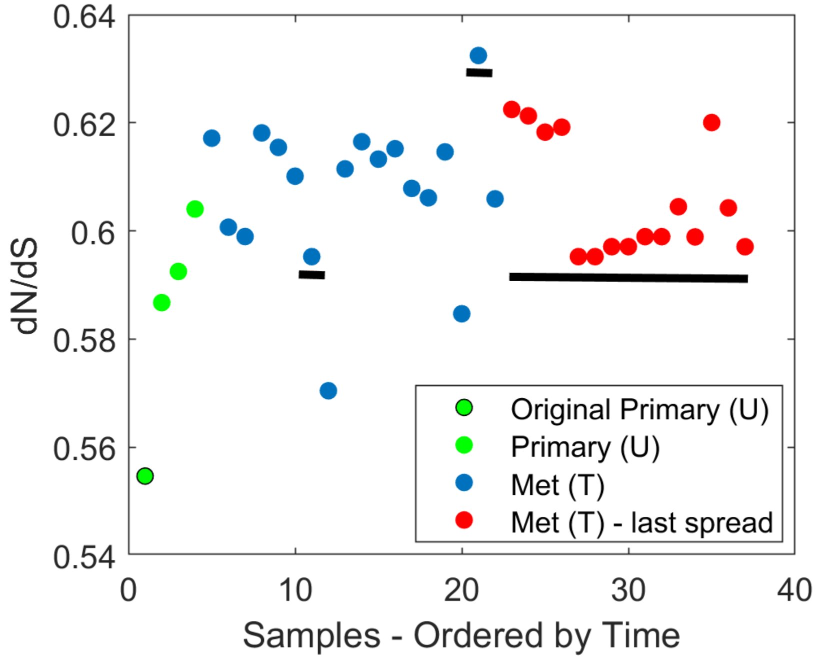

Figure S16: **Analysis of a melanoma patient**. *dN/dS* values of **37 samples** of the melanoma patient (age 67) with before (green) and trough immune-checkpoint-blocker (**ICB**) treatment, including: original (n=1) and **pre-treatment** (n=3) primary samples, **on-treatment** (n=18, blue) and **post-treatment progression** (n=15, red), spanning about 9 years in total (and about 6 years from ICB onset). The lineage that was responsible for the last post-treatment metastatic burst is marked (thick black lines) according to the phylogenetic analysis in the original study. **Note that** although the *dN/dS* values are much lower from neutral regime (expected from the huge number of deleterious passenger mutations in Melanoma) the lineage that eventually resisted therapy and led to the last burst of metastases came from sample with the highest dN/dS values (i.e., as close as possible to neutral regime for this cancer type).

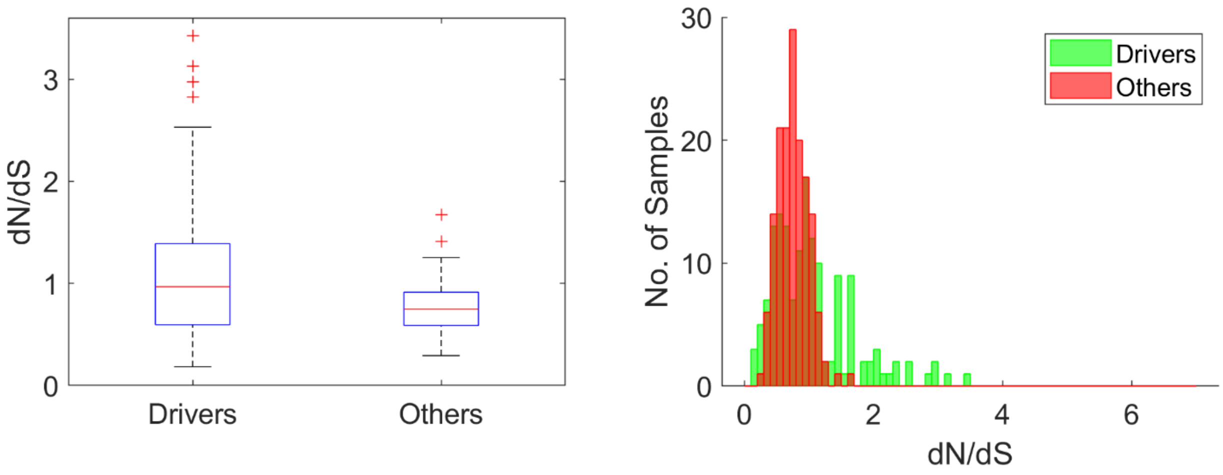

Figure S17: **Evaluation of dN/dS in driver genes in the multiple myeloma cohort.** The distribution of dN/dS across samples, when evaluated for the set of 585 driver genes, as defined by COSMIC is shown vs. the distribution of dN/dS across samples for the set of all other genes in the human genome. The distributions are provided as boxplot (*left*) and as histogram (*right*). Note the elevated ratio of dN/dS in driver genes. Box plots denote the median (red) and the edges of the box are the 25th and 75th percentiles, with whiskers extending to the most extreme data points.

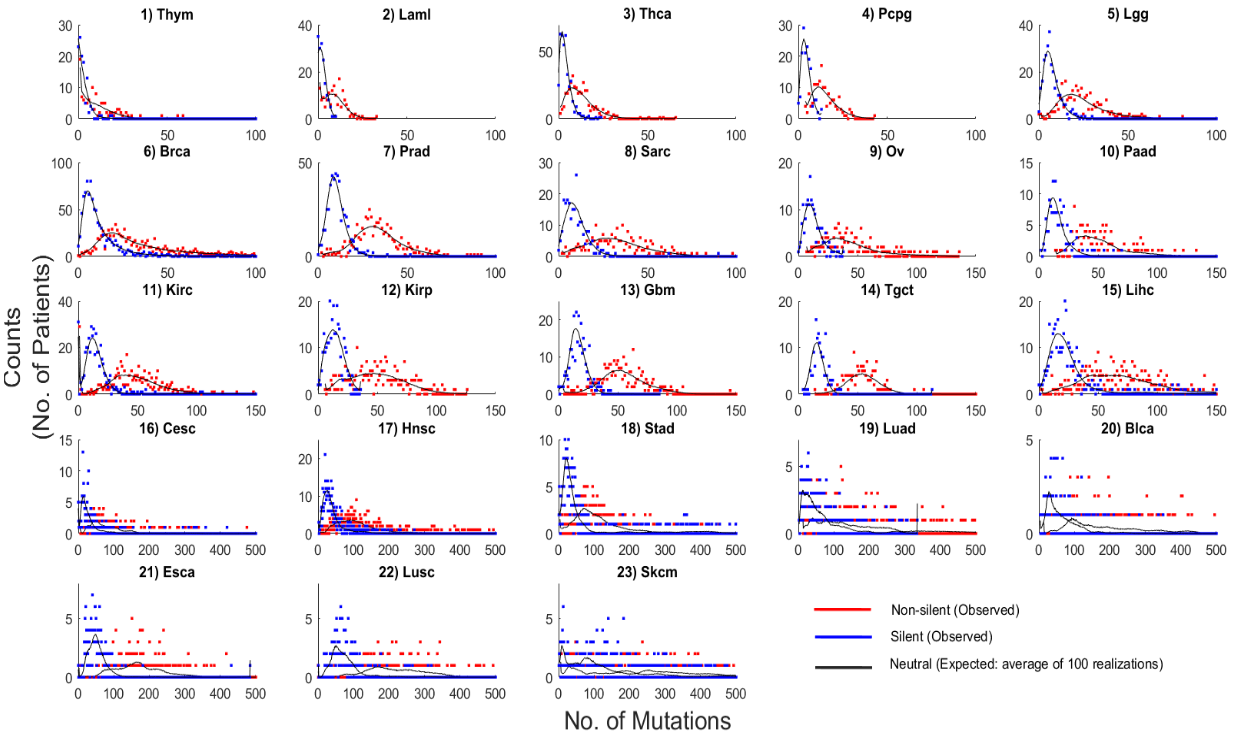

Figure S18: **Observed vs. expected distributions of *N* and *S* mutations across cancer types.** For Each of the 23 cancer types from TCGA database (ordered from the low to high mutation burden), the observed distributions of *N* (red dots) and *S* (blue dots) mutations, and the expected distributions from an equivalent neutral Poisson model of the pan-cancer data (black curves), are shown. To produce the black curves, an optimal reference neutral model equivalent to the pan-cancer data, representative of the diversity across patients was generated. First, we assessed the total number of mutations *M* (=*N*+*S*) in each tumor (i.e., patient/sample). Then, we generated two matrices ($\lambda^N_{s,g}$, $\lambda^S_{s,g}$) of the expected number of *N* and *S* mutations in each gene (*g*) and each sample (s), such that $\lambda^N_{s,g} = M_s \; x \; L_g \; x \; R_g^N$, and $\lambda^S_{s,g} = M_s \; x \; L_g \; x \; R_g^S$; where M_s is the total number of mutations in a sample, L_g is the normalized length a gene ($\sum_g L_g$=1) and R_g^N, R_g^S are the normalized number *N* and *S* sites in each gene (R_g^N+R_g^S=1). Using $\lambda^N_{s,g}$, $\lambda^S_{s,g}$ we generated the expected number of *N* and *S* mutations under neutrality in each sample, using the fact that for a Poisson process $\lambda_s = \sum_g \lambda_{s,g}$. This procedure produced *dN/dS* distributions across patients and cancer types that are highly comparable to the observed distributions, reflecting the exact diversity of λ across patients and cancers. The expected count distributions (black curves) were estimated from the average over the 1000 realizations of the neutral model.

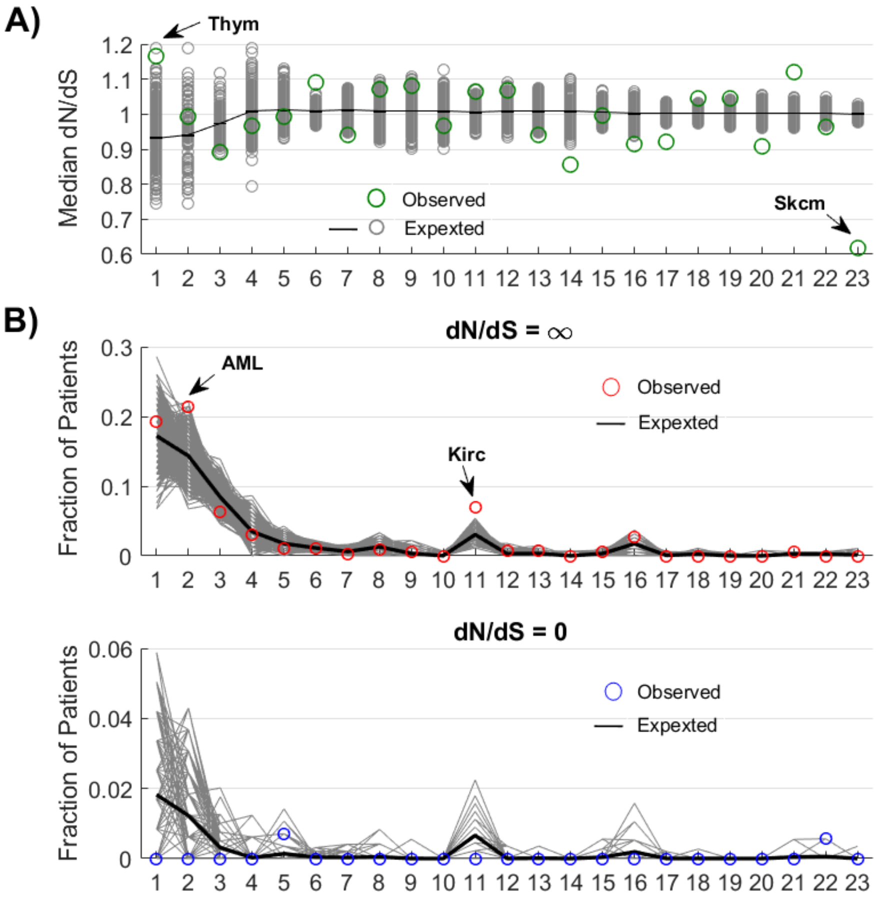

Figure S19: **Observed vs. expected statistics of distribution of *dN/dS***. **A)** The observed medians of the distributions of *dN/dS* in each cancer type (green circles, same order as in **Fig. S18**) and the medians obtained from 1000 different realizations (gray circles) of the neutral Poisson expectations. The mean of the median *dN/dS* across realizations is shown (black curve). A deviation of the mean *dN/dS* from neutrality is observed in low mutational burdens ((where *dN/dS<1*). **B**) The fraction dN/dS = ∞ (*top*) and dN/dS = 0 (*bottom*), in the cancer data (colored circles) and across neutral Poisson realizations of the data (gray lines) and the mean across realizations (black curve). The fundamental larger number of *N* sites vs. *S* sites, leads in low mutational burden to more cases of *dN/dS*=∞ than cases with *dN/dS=0*. Because such cases are excluded from analysis of *dN/dS* distribution statistics, they render the theoretical prediction biased toward negative selection, as shown in (A). The deviation is apparent in cancers where patients typically exhibit **about 10 somatic mutations** in the primary tumor genome (e.g., Thymoma and AML), concluding the minimal threshold below which patients/samples should be excluded from analysis in the current study, to avoid sampling bias.

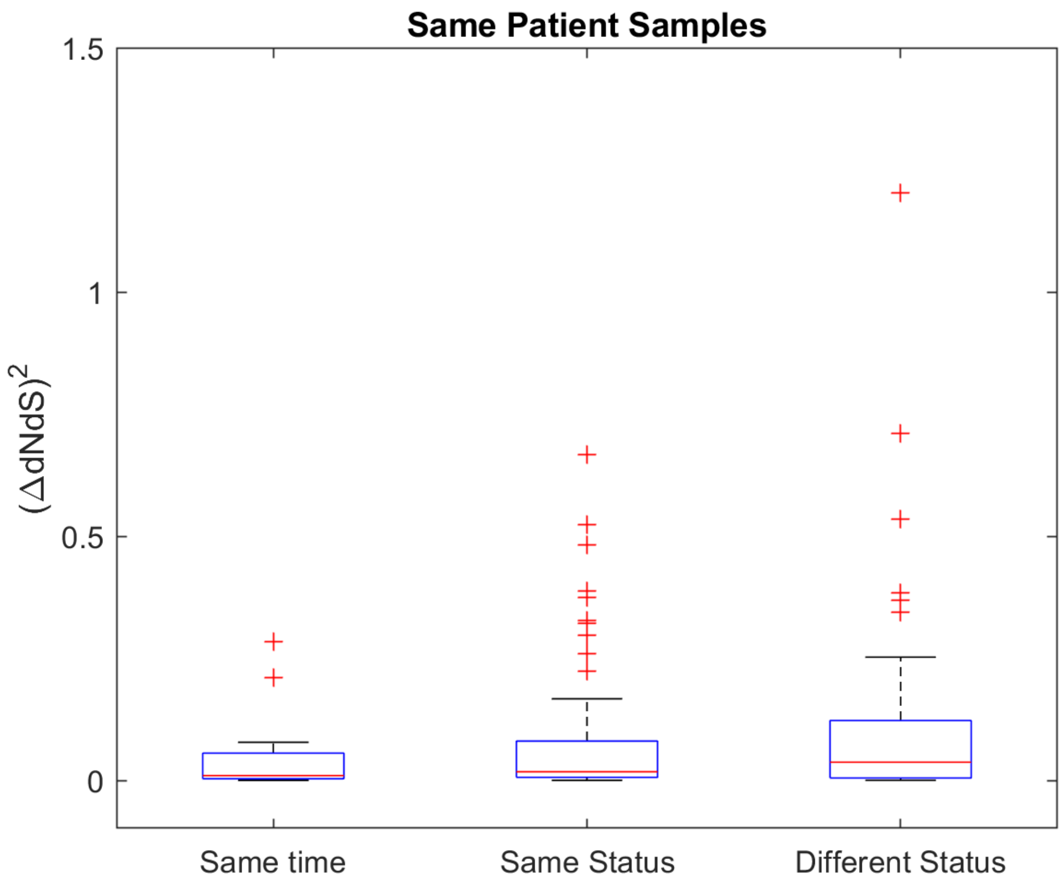

Figure S20: **Error estimation of *dN/dS* in the multiple myeloma cohort.** The squared difference in *dN/dS* between any two samples of the same patient are shown separately for samples taken at the same time (i.e., similar biopsy, sequenced separately), samples of the same status (e.g., pre-treatment or recurred) taken at different times, and samples of different status and different times. The flat distribution for samples taken at the same time (and obviously same status) indicates low error in the evaluation of *dN/dS.* Note that the larger differences in the other cases (i.e., same status but different times, and different status and times) do not reflect merely error, because a change in *dN/dS* could result from the evolution of the tumor in each patient; nonetheless, but these distributions are relatively narrow due to the general invariance and stability of *dN/dS* values in a patient. Box plots denote the median (red) and the edges of the box are the 25th and 75th percentiles, with whiskers extending to the most extreme data points.

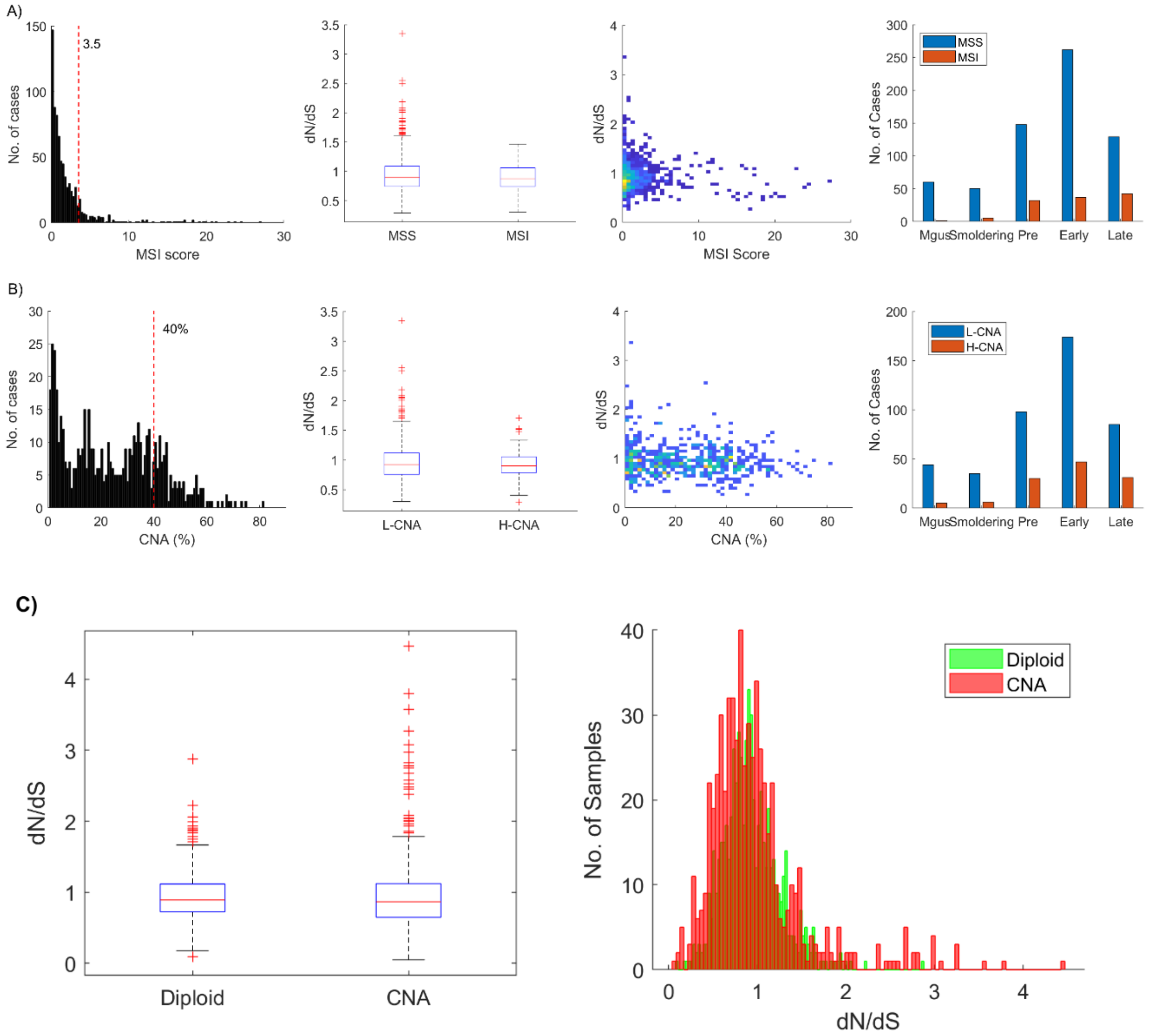

Figure S21: **Stability of *dN/dS* with respect to MSI and CNA in the multiple myeloma cohort. A**) The distribution of MSI scores, whereby score > 3.5 denote unstable (MSI) tumor genomes and score below this threshold correspond to stable (MSS) tumor genomes (***left***). Box plots of the distributions of dN/dS in MSS and MSI genomes are indistinguishable (***middle-left***). Heatmap shows dN/dS values as function of MSI score (***middle-right***). MSI accumulate with disease progression (***right***). **B**) Similar analysis with respect to the percentage of genes affected by CNA, indicating the stability of dN/dS with respect to CNA. High (H) CNA are cases with CNA>40% corresponding to the upper 25 percentile and low (L) CAN are cases with CNA<40%. **C**) dN/dS distribution in diploid regions and regions affected by CNA, shown as boxplot (***left***) and as histograms (***right***), also indicating that dN/dS metric is insensitive to the genome stability status. Box plots denote the median (red) and the edges of the box are the 25th and 75th percentiles, with whiskers extending to the most extreme data points.

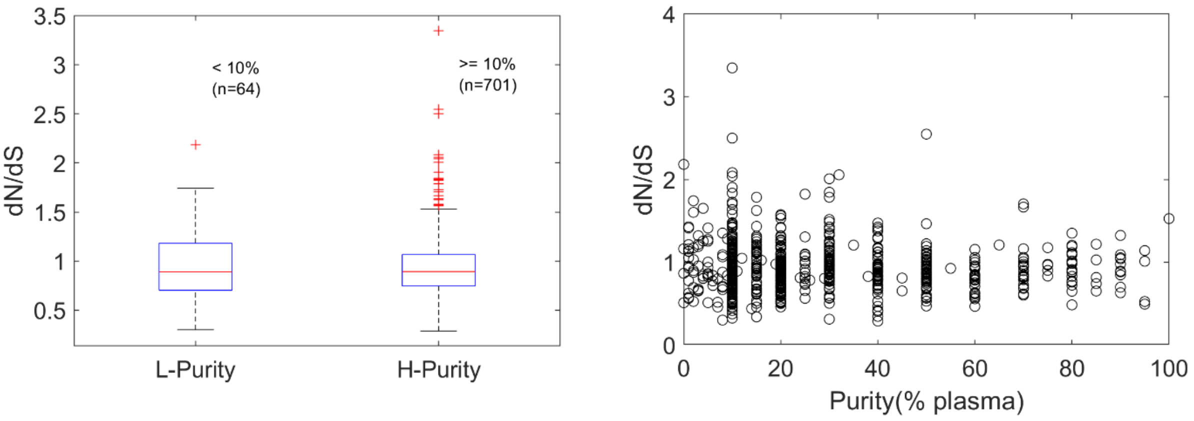

Figure S22: **Stability of *dN/dS* with respect to tumor purity in the multiple myeloma cohort.** The distributions of dN/dS in low (L) tumor purity samples (cancer plasma cells < 10%) before enrichment by CD138 (and sequencing) and in high (H) tumor purity, indicating that dN/dS is largely unaffected by the pre-enrichment tumor purity level (***left***). Box plots denote the median (red) and the edges of the box are the 25th and 75th percentiles, with whiskers extending to the most extreme data points. The corresponding scatter plot between dN/dS and purity level (***right***).
