# Supplementary material for: Genome-level selection in tumors as a universal marker of resistance to therapy
Source: Nat Commun. 2025 Jul 16;16:6535. doi: 10.1038/s41467-025-61709-x (PMC12263839; doi:10.1038/s41467-025-61709-x)
Supplement: Supplementary file 3 — Description of Additional Supplementary Files [file 41467_2025_61709_MOESM3_ESM.pdf]

Description of Additional Supplementary Files

Supplementary Software 1: Supplementary Software is provided in the file CODE.zip. The file contains the mutation and clinical data used for analysis, as well as the MATLAB analysis scripts, which read and process the data and reproduce the results.

The data and files are organized into folders; each represents a dataset that was analysed in this study.

To use the software and reproduce the results, extract CODE.zip and follow the instructions in the READ_ME file.
