# Supplementary figures and images for: Genome-level selection in tumors as a universal marker of resistance to therapy
Source: Nat Commun. 2025 Jul 16;16:6535. doi: 10.1038/s41467-025-61709-x (PMC12263839; doi:10.1038/s41467-025-61709-x)

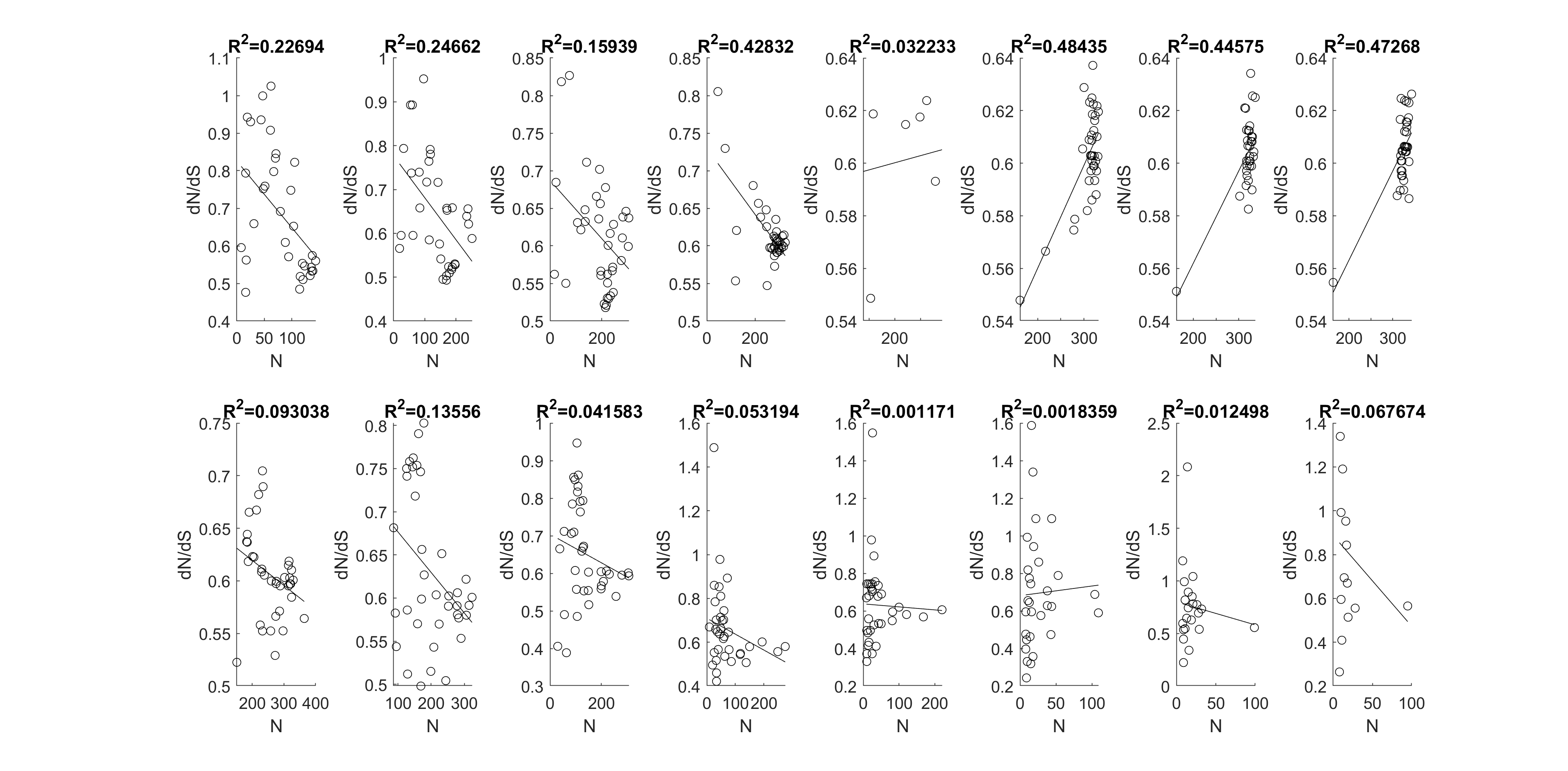

Supplement: Supplementary file 4 — Supplementary Software 1 [file 41467_2025_61709_MOESM4_ESM.zip › CODE/Melanoma/nat_med_2021/dNdS_N_corr.tif]
