# Supplementary material for: Genome-level selection in tumors as a universal marker of resistance to therapy
Source: Nat Commun. 2025 Jul 16;16:6535. doi: 10.1038/s41467-025-61709-x (PMC12263839; doi:10.1038/s41467-025-61709-x)
Supplement: Supplementary file 5 — Reporting Summary [file 41467_2025_61709_MOESM5_ESM.pdf]

nature portfolio

Corresponding author(s): Erez Persi, Ariosto Silva, Eugene Koonin

Last updated by author(s): Jun 17, 2025

Reporting Summary

Nature Portfolio wishes to improve the reproducibility of the work that we publish. This form provides structure for consistency and transparency in reporting. For further information on Nature Portfolio policies, see our Editorial Policies and the Editorial Policy Checklist.

Statistics

For all statistical analyses, confirm that the following items are present in the figure legend, table legend, main text, or Methods section.

n/a	Confirmed	
☐	☒	The exact sample size (*n*) for each experimental group/condition, given as a discrete number and unit of measurement
☐	☒	A statement on whether measurements were taken from distinct samples or whether the same sample was measured repeatedly
☐	☒	The statistical test(s) used AND whether they are one- or two-sided<br>*Only common tests should be described solely by name; describe more complex techniques in the Methods section.*
☐	☒	A description of all covariates tested
☐	☒	A description of any assumptions or corrections, such as tests of normality and adjustment for multiple comparisons
☐	☒	A full description of the statistical parameters including central tendency (e.g. means) or other basic estimates (e.g. regression coefficient) AND variation (e.g. standard deviation) or associated estimates of uncertainty (e.g. confidence intervals)
☐	☒	For null hypothesis testing, the test statistic (e.g. *F*, *t*, *r*) with confidence intervals, effect sizes, degrees of freedom and *P* value noted<br>*Give P values as exact values whenever suitable.*
☒	☐	For Bayesian analysis, information on the choice of priors and Markov chain Monte Carlo settings
☒	☐	For hierarchical and complex designs, identification of the appropriate level for tests and full reporting of outcomes
☐	☒	Estimates of effect sizes (e.g. Cohen's *d*, Pearson's *r*), indicating how they were calculated

Our web collection on statistics for biologists contains articles on many of the points above.

Software and code

Policy information about availability of computer code

Data collection	All data is collected from the supplementary data of the respective analyzed published datasets (see Data and Code availability). The data for the original Multiple Myeloma is described in Methods and available as described in Data and Code availability Statements. All the processed source data used for the analysis is also included in the Supplementary Software 1 (Code.zip)
Data analysis	All the analyses were done in MATLAB and the code to reproduce the results and figure is provided as Supplementary Software 1 (Code.zip)

For manuscripts utilizing custom algorithms or software that are central to the research but not yet described in published literature, software must be made available to editors and reviewers. We strongly encourage code deposition in a community repository (e.g. GitHub). See the Nature Portfolio guidelines for submitting code & software for further information.

Data

Policy information about availability of data

All manuscripts must include a data availability statement. This statement should provide the following information, where applicable:

- Accession codes, unique identifiers, or web links for publicly available datasets
- A description of any restrictions on data availability
- For clinical datasets or third party data, please ensure that the statement adheres to our policy

All the data used for analysis is public, as provided by each study analyzed (see Methods, datasets, and Data availability Statement). For the original multiple myeloma cohort:

• WES: The processed MAF file for multiple myeloma samples collected at H. Lee Moffitt Cancer Center & Research Institute with mutations annotated can be downloaded from the following folder in Synapse, https://doi.org/10.7303/syn53268838.
• Access to Raw Molecular Data: The molecular data used in this research was generated through private funding by Aster Insights (www.asterinsights.com) in collaboration with the Oncology Research Information Exchange Network (ORIEN, www.oriencancer.org). Requests for access to the raw data (FASTQ/BAM) used in this study can be submitted to the corresponding author (A.S.S.) and at https://researchdatarequest.orienavatar.com/.
* All the source data used for analysis and the code to reproduce the main results and figure are provided in Supplementary Software - Code.Zip (follow the READ_ME file). See Data and Code availability statements.

Research involving human participants, their data, or biological material

Policy information about studies with human participants or human data. See also policy information about sex, gender (identity/presentation), and sexual orientation and race, ethnicity and racism.

Reporting on sex and gender	Provided in the source data of the published datasets. For the multiple myeloma samples, Sex (and race and ethnicity) was not considered in the study design and was based on all available patients. Self-reporting from electronic health records, was provided to us by an honest broker, with balanced demographic of 56% male and 44% female. See Data and Code availability as well as Supplementary Software.
Reporting on race, ethnicity, or other socially relevant groupings	Provided in the source data of the published datasets. For the multiple myeloma samples, Sex (and race and ethnicity) was not considered in the study design and was based on all available patients. Self-reporting from electronic health records, was provided to us by an honest broker, with balanced demographic of 56% male and 44% female. See Data and Code availability as well as Supplementary Software.
Population characteristics	*Describe the covariate-relevant population characteristics of the human research participants (e.g. age, genotypic information, past and current diagnosis and treatment categories). If you filled out the behavioural & social sciences study design questions and have nothing to add here, write "See above."*
Recruitment	As described in Methods, for the multiple myeloma patients
Ethics oversight	As described in Methods (datasets)

Note that full information on the approval of the study protocol must also be provided in the manuscript.

Field-specific reporting

Please select the one below that is the best fit for your research. If you are not sure, read the appropriate sections before making your selection.

☒ Life sciences ☐ Behavioural & social sciences ☐ Ecological, evolutionary & environmental sciences

For a reference copy of the document with all sections, see nature.com/documents/nr-reporting-summary-flat.pdf

Life sciences study design

All studies must disclose on these points even when the disclosure is negative.

Sample size	All data is as described in and provided by the respective studies. All the data (and analysis code) is now available in Code.zip
Data exclusions	Samples with too small number of mutations - as described in METHODS.
Replication	*Describe the measures taken to verify the reproducibility of the experimental findings. If all attempts at replication were successful, confirm this OR if there are any findings that were not replicated or cannot be reproduced, note this and describe why.*
Randomization	*Describe how samples/organisms/participants were allocated into experimental groups. If allocation was not random, describe how covariates were controlled OR if this is not relevant to your study, explain why.*
Blinding	*Describe whether the investigators were blinded to group allocation during data collection and/or analysis. If blinding was not possible, describe why OR explain why blinding was not relevant to your study.*

Reporting for specific materials, systems and methods

We require information from authors about some types of materials, experimental systems and methods used in many studies. Here, indicate whether each material, system or method listed is relevant to your study. If you are not sure if a list item applies to your research, read the appropriate section before selecting a response.

Materials & experimental systems

n/a	Involved in the study
☒	☐ Antibodies
☒	☐ Eukaryotic cell lines
☒	☐ Palaeontology and archaeology
☒	☐ Animals and other organisms
☐	☒ Clinical data
☒	☐ Dual use research of concern
☒	☐ Plants

Methods

n/a	Involved in the study
☒	☐ ChIP-seq
☒	☐ Flow cytometry
☒	☐ MRI-based neuroimaging

Clinical data

Policy information about clinical studies

All manuscripts should comply with the ICMJE guidelines for publication of clinical research and a completed CONSORT checklist must be included with all submissions.

Clinical trial registration	Clinical data used for analysis is as provided by the respective studies and as collected for the original MM cohort (See METHODS)
Study protocol	As described in METHODS and Data avauilability
Data collection	Overall Survival of patients
Outcomes	Censored data (Alive, Dead)

Plants

Seed stocks	*Report on the source of all seed stocks or other plant material used. If applicable, state the seed stock centre and catalogue number. If plant specimens were collected from the field, describe the collection location, date and sampling procedures.*
Novel plant genotypes	*Describe the methods by which all novel plant genotypes were produced. This includes those generated by transgenic approaches, gene editing, chemical/radiation-based mutagenesis and hybridization. For transgenic lines, describe the transformation method, the number of independent lines analyzed and the generation upon which experiments were performed. For gene-edited lines, describe the editor used, the endogenous sequence targeted for editing, the targeting guide RNA sequence (if applicable) and how the editor was applied.*
Authentication	*Describe any authentication procedures for each seed stock used or novel genotype generated. Describe any experiments used to assess the effect of a mutation and, where applicable, how potential secondary effects (e.g. second site T-DNA insertions, mosiacism, off-target gene editing) were examined.*
