# Supplementary material for: Genome-level selection in tumors as a universal marker of resistance to therapy
Source: Nat Commun. 2025 Jul 16;16:6535. doi: 10.1038/s41467-025-61709-x (PMC12263839; doi:10.1038/s41467-025-61709-x)
Supplement: Supplementary file 4 — Supplementary Software 1 [file 41467_2025_61709_MOESM4_ESM.zip › CODE/MM_MOFFITT/READ_ME_multi_response.docx]

This relates to the data in ‘MULTI_response.mat’ file. This has the updated data structure MULTI with four new columns described below:

- **Response**: In this field you will find a table of clinical response between every consecutive pairs of biopsies, first column is number of days and second column is percent tumor burden.
- **Inflection Points**: Using the data in the above Response table, we estimated points of inflection, also saved as tables similar to the Response field.
- **ResponsePlot**: This field has the plot saved as an image matrix that you can output to the console using the command imshow(MULTI(i).ResponsePlot{j}) for the i^th^ patient’s response between j^th^ and (j+1)^th^ biopsy.
- **Classification**: This field is the call for response. We structured it in a way so that it is as descriptive as possible. In between the two biopsies, if a patient goes from responder to non-responder to responder then it is annotated as “Responder_NonResponder_Responder” in this field. So every time there is a change in response call between consecutive points of inflection we included an annotation for that. The interesting part is what is happening close to the first biopsy and second biopsy - then one may split this string using strsplit(x,’_’) and use the first and last element.
