# Supplementary material for: Genome-level selection in tumors as a universal marker of resistance to therapy
Source: Nat Commun. 2025 Jul 16;16:6535. doi: 10.1038/s41467-025-61709-x (PMC12263839; doi:10.1038/s41467-025-61709-x)
Supplement: Supplementary file 4 — Supplementary Software 1 [file 41467_2025_61709_MOESM4_ESM.zip › CODE/MM_MOFFITT/Signatures/LRMM.pdf]

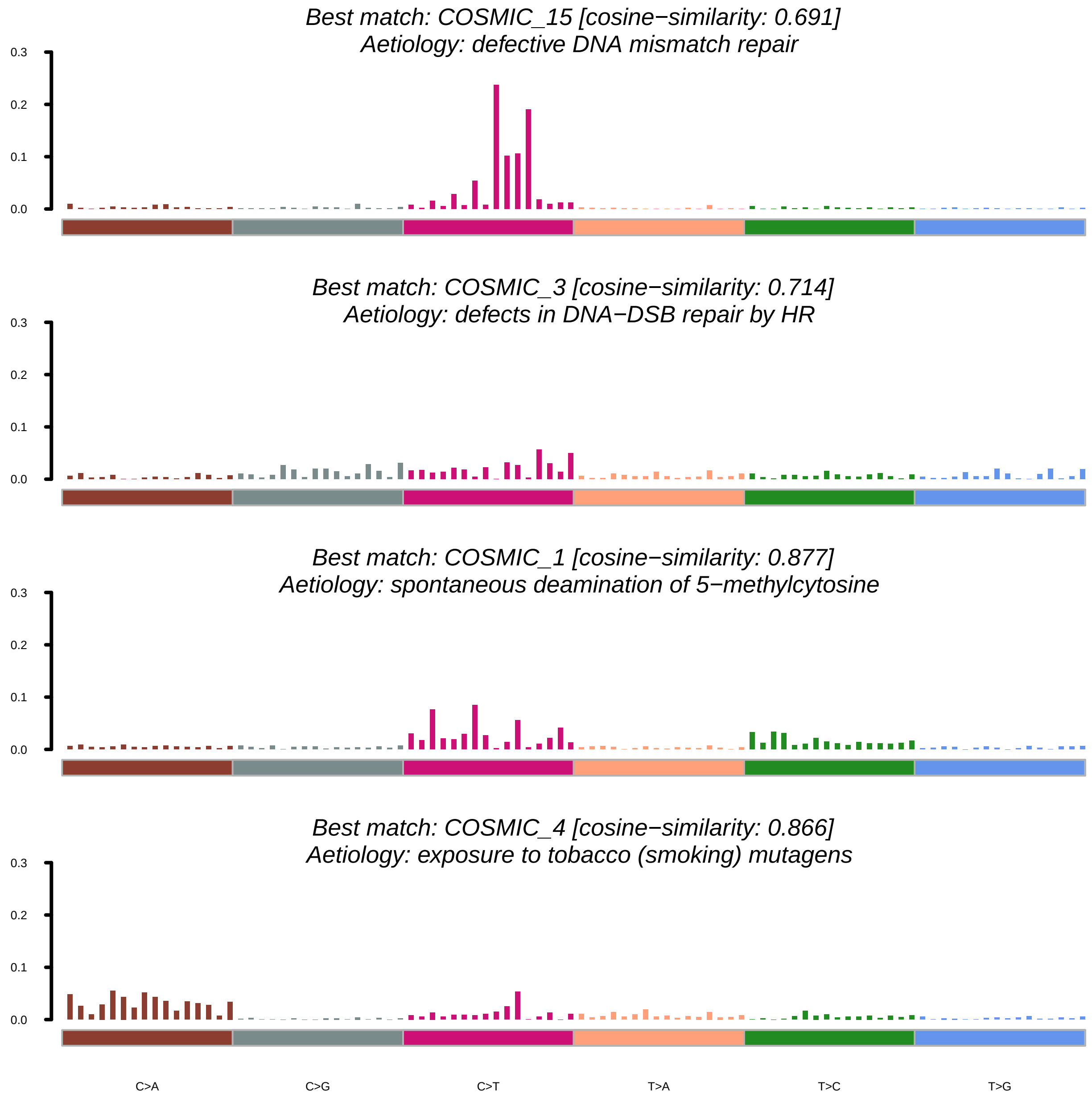

Best match: COSMIC_15 [cosine−similarity: 0.691]
Aetiology: defective DNA mismatch repair
Best match: COSMIC_3 [cosine−similarity: 0.714]
Aetiology: defects in DNA−DSB repair by HR
Best match: COSMIC_1 [cosine−similarity: 0.877]
Aetiology: spontaneous deamination of 5−methylcytosine
Best match: COSMIC_4 [cosine−similarity: 0.866]
Aetiology: exposure to tobacco (smoking) mutagens
0.0
0.1
0.2
0.3
C>A
C>G
C>T
T>A
T>C
T>G

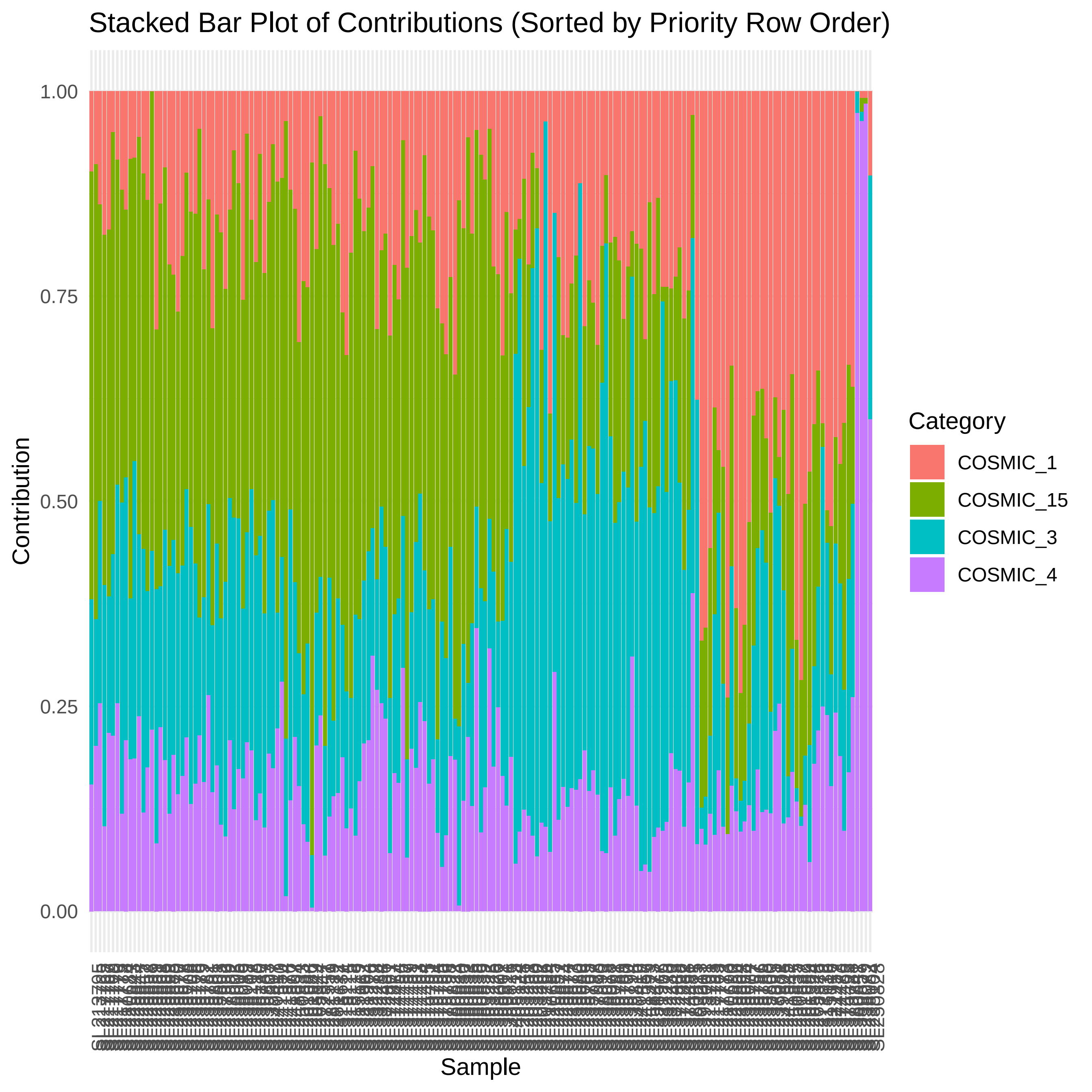
Stacked Bar Plot of Contributions (Sorted by Priority Row Order)
Contribution
1.00
0.75
0.50
0.25
0.00
Sample
Category
COSMIC_1
COSMIC_15
COSMIC_3
COSMIC_4

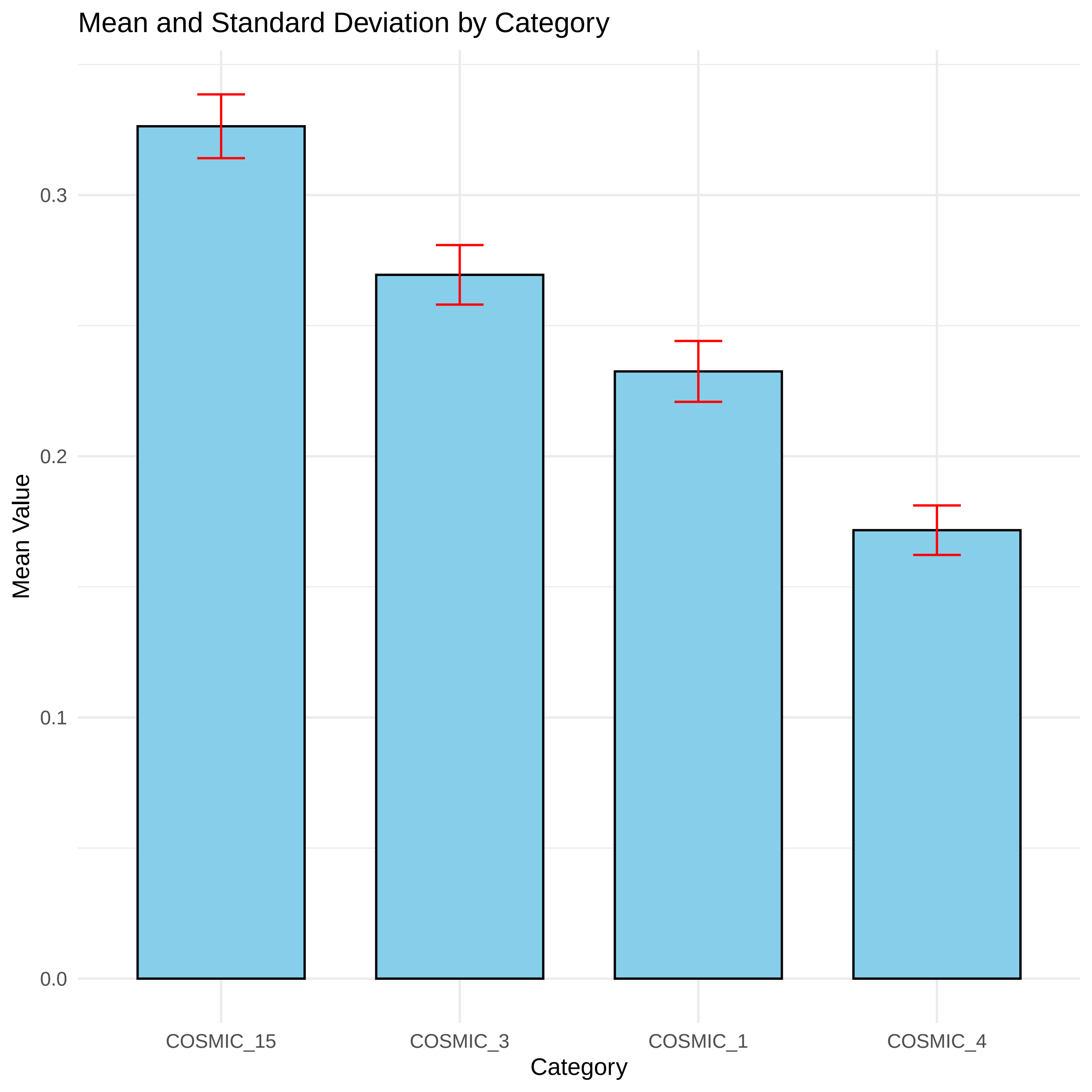

Mean and Standard Deviation by Category
Mean Value
0.3
0.2
0.1
0.0
COSMIC_15
COSMIC_3
COSMIC_1
COSMIC_4
Category
