# Supplementary material for: Genome-level selection in tumors as a universal marker of resistance to therapy
Source: Nat Commun. 2025 Jul 16;16:6535. doi: 10.1038/s41467-025-61709-x (PMC12263839; doi:10.1038/s41467-025-61709-x)
Supplement: Supplementary file 4 — Supplementary Software 1 [file 41467_2025_61709_MOESM4_ESM.zip › CODE/MM_MOFFITT/Signatures/MGUS.pdf]

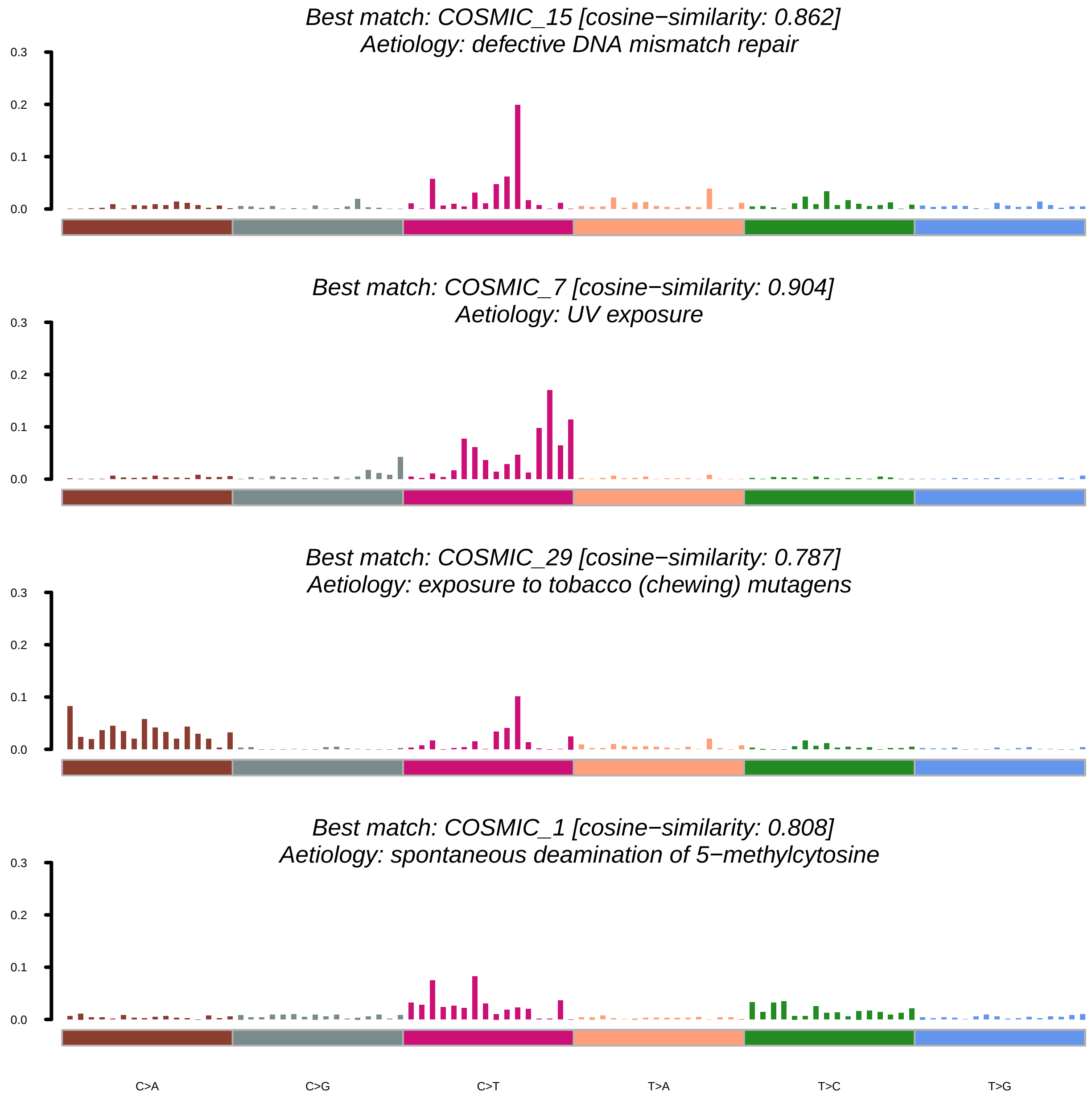

Best match: COSMIC_15 [cosine−similarity: 0.862]
Aetiology: defective DNA mismatch repair
Best match: COSMIC_7 [cosine−similarity: 0.904]
Aetiology: UV exposure
Best match: COSMIC_29 [cosine−similarity: 0.787]
Aetiology: exposure to tobacco (chewing) mutagens
Best match: COSMIC_1 [cosine−similarity: 0.808]
Aetiology: spontaneous deamination of 5−methylcytosine
0.0
0.1
0.2
0.3
C>A
C>G
C>T
T>A
T>C
T>G

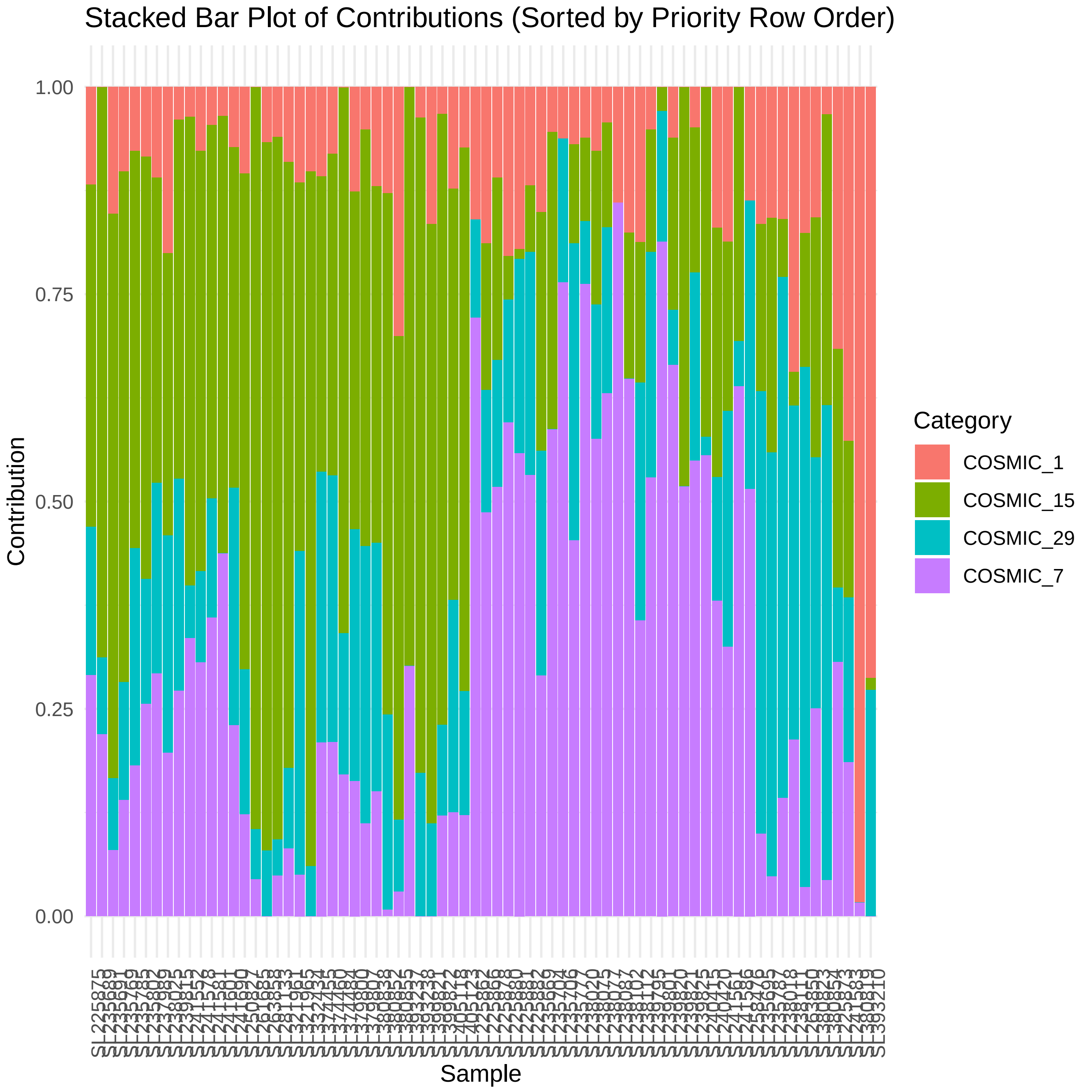
Stacked Bar Plot of Contributions (Sorted by Priority Row Order)
1.00
0.75
0.50
0.25
0.00
Contribution
Sample
Category
COSMIC_1
COSMIC_15
COSMIC_29
COSMIC_7

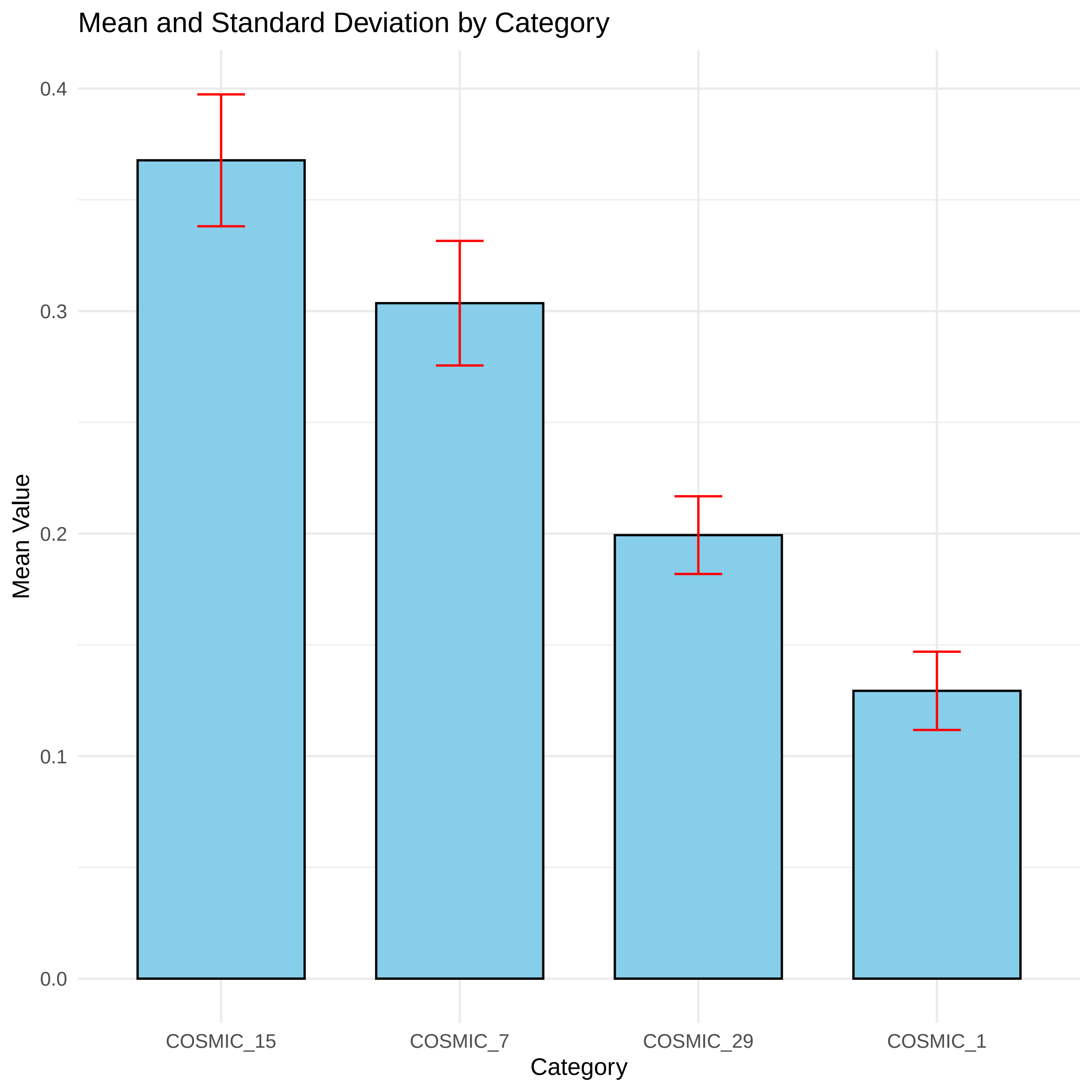
Mean and Standard Deviation by Category
0.4
0.3
0.2
0.1
0.0
Mean Value
COSMIC_15
COSMIC_7
COSMIC_29
COSMIC_1
Category
