# Supplementary material for: Genome-level selection in tumors as a universal marker of resistance to therapy
Source: Nat Commun. 2025 Jul 16;16:6535. doi: 10.1038/s41467-025-61709-x (PMC12263839; doi:10.1038/s41467-025-61709-x)
Supplement: Supplementary file 4 — Supplementary Software 1 [file 41467_2025_61709_MOESM4_ESM.zip › CODE/MM_MOFFITT/Signatures/NDMM.pdf]

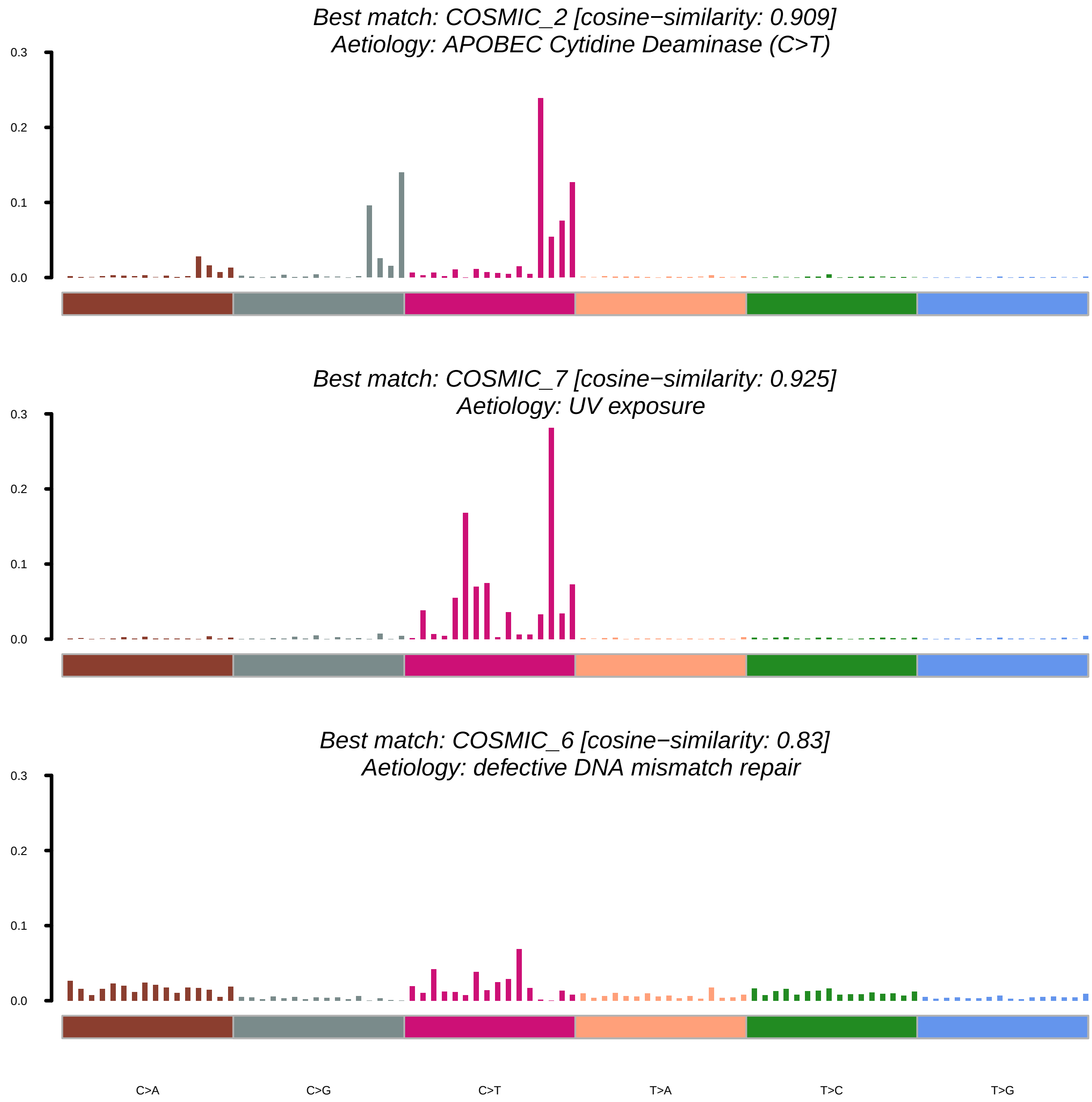

Best match: COSMIC_2 [cosine−similarity: 0.909]
Aetiology: APOBEC Cytidine Deaminase (C>T)
0.3
0.2
0.1
0.0
Best match: COSMIC_7 [cosine−similarity: 0.925]
Aetiology: UV exposure
0.3
0.2
0.1
0.0
Best match: COSMIC_6 [cosine−similarity: 0.83]
Aetiology: defective DNA mismatch repair
0.3
0.2
0.1
0.0
C>A
C>G
C>T
T>A
T>C
T>G

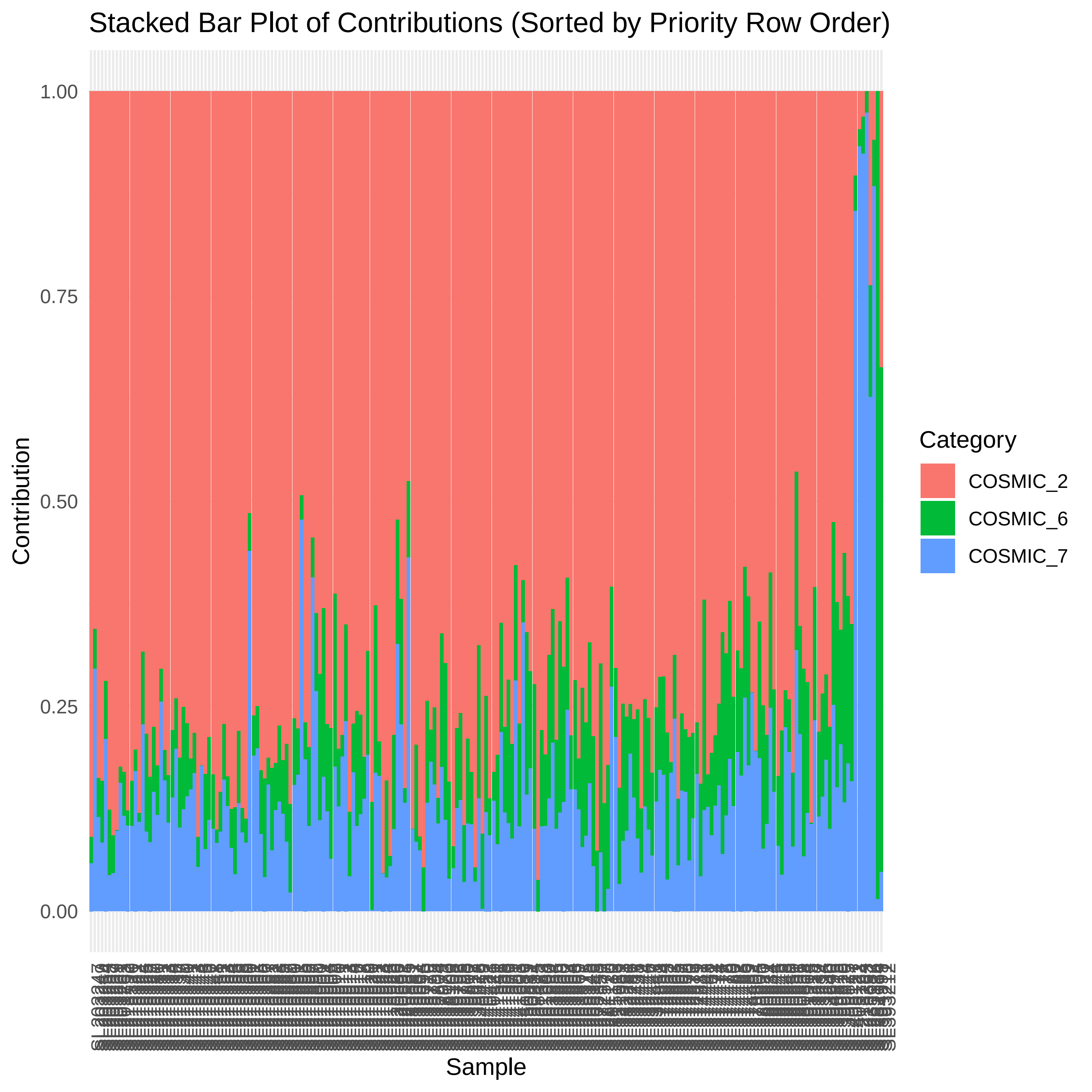
Stacked Bar Plot of Contributions (Sorted by Priority Row Order)
Contribution
1.00
0.75
0.50
0.25
0.00
Sample
Category
COSMIC_2
COSMIC_6
COSMIC_7

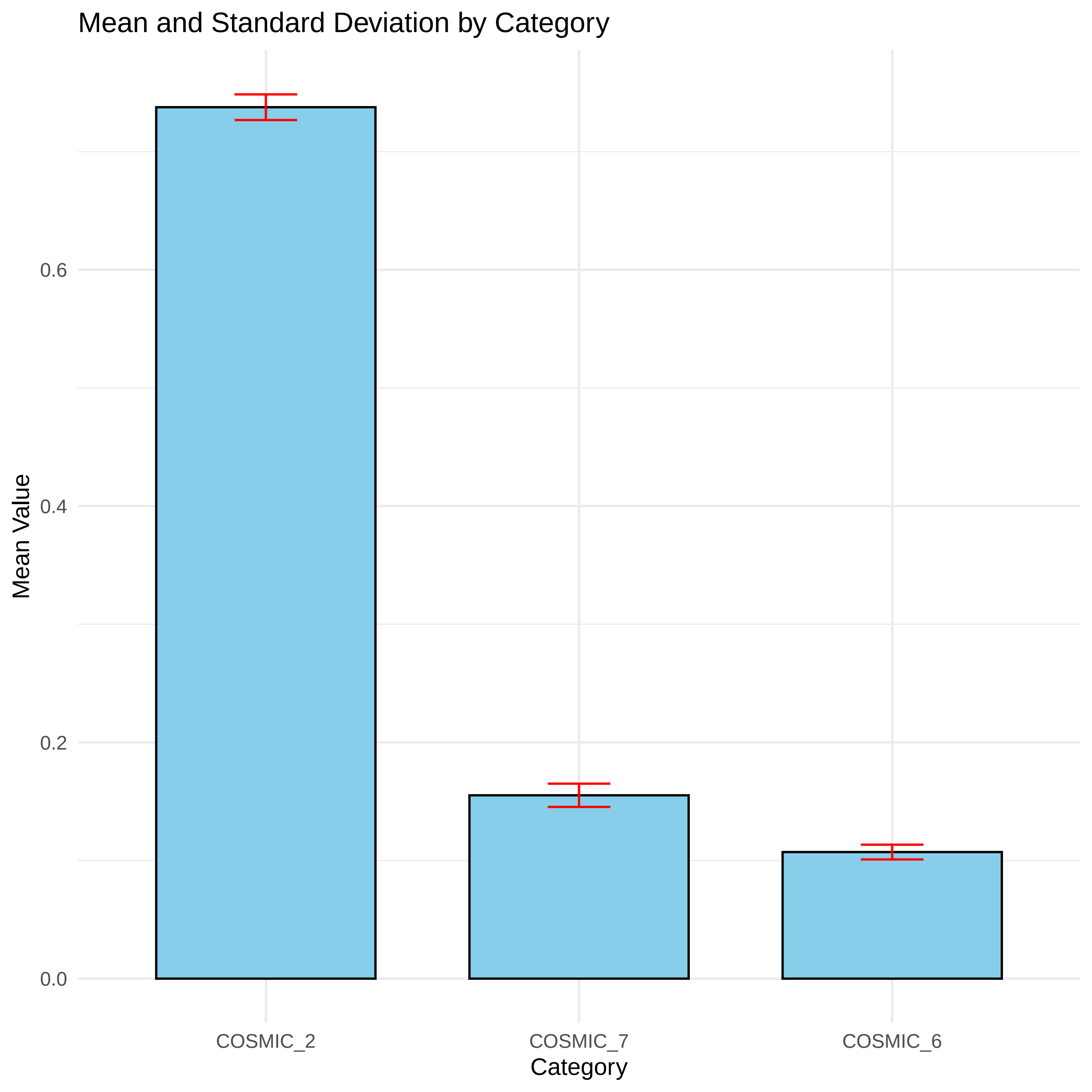
Mean and Standard Deviation by Category
Mean Value
0.0
0.2
0.4
0.6
COSMIC_2
COSMIC_7
COSMIC_6
Category
