# Supplementary material for: Genome-level selection in tumors as a universal marker of resistance to therapy
Source: Nat Commun. 2025 Jul 16;16:6535. doi: 10.1038/s41467-025-61709-x (PMC12263839; doi:10.1038/s41467-025-61709-x)
Supplement: Supplementary file 4 — Supplementary Software 1 [file 41467_2025_61709_MOESM4_ESM.zip › CODE/READ_ME.docx]

How to reproduce the Results reported in “**Genome Level Selection in Tumors as a Universal Marker of Resistance to Treatment**” by Persi et al.

To reproduce the main results and figures of each analyzed study, run MATLAB, go to the folder of each study (e.g., ALL folder) and execute the file MAIN.m and additional m-files (such as PLOT.m and PrePost.m if exist) to produce additional plots. If the file MAIN.m does not exist, run the file ‘estimate_dNdS.m’.

Each folder contains the relevant source mutational and clinical data as provided by the supplementary information of each respective study, as well as processed data in ‘.mat’ format (for ease of use). Inside each .m-file there are detailed explanations for most line codes - to make the code easy to follow and understand. Typically, the execution times of these files range between a few seconds up to a couple of minutes (depending on the size of the analyzed dataset).

To reproduce the results of the statistical analyses that compare linear regressions models using ANOVA, run the file ‘compare_linear_models.m’ in the relevant folder(s).

To reproduce the results of survival analysis, run the file ‘estimate_Survival.m’ in the relevant folder(s).

**Additional Notes**:

1) the folder for the analysis of the 5 breast cancer cohort (c.f., Figure 3) are in the ‘Breast’ folder (run ‘Breast_analysis.m’ to reproduce the figure). There are 5 subfolders with the respective code for each of the 5 analyzed studies.

2) the folder for the analysis of the original MM cohort (c.f., Figure 4) contain additional m-files relevant to reproduce the analysis – the main files include ‘PrePost.m’, ‘Treated.m’, ‘Untreated.m’, as well as ‘clonal_vs_subclonal_dNdS.m’, ‘drivers_dNdS.m’. In each subfolder (e.g., CNA, MSI) please find the relevant data and m-files to reproduce the results

3) to reproduce the analysis of single patients (cf. Figure 6) run the files starting with ‘single_.m’ in the relevant folder(s)
